# Link-Prediction Enhanced Consensus Clustering for Complex Networks

**DOI:** 10.1371/journal.pone.0153384

**Published:** 2016-05-20

**Authors:** Matthew Burgess, Eytan Adar, Michael Cafarella

**Affiliations:** 1 Computer Science & Engineering, University of Michigan, Ann Arbor, MI, United States of America; 2 School of Information, University of Michigan, Ann Arbor, MI, United States of America; University of Vermont, UNITED STATES

## Abstract

Many real networks that are collected or inferred from data are incomplete due to missing edges. Missing edges can be inherent to the dataset (Facebook friend links will never be complete) or the result of sampling (one may only have access to a portion of the data). The consequence is that downstream analyses that “consume” the network will often yield less accurate results than if the edges were complete. Community detection algorithms, in particular, often suffer when critical intra-community edges are missing. We propose a novel consensus clustering algorithm to enhance community detection on incomplete networks. Our framework utilizes existing community detection algorithms that process networks imputed by our link prediction based sampling algorithm and merges their multiple partitions into a final consensus output. On average our method boosts performance of existing algorithms by 7% on artificial data and 17% on ego networks collected from Facebook.

## Introduction

Many types of complex networks exhibit community structure: groups of highly connected nodes. Communities or clusters often reflect nodes that share similar characteristics or functions. For instance, communities in social networks can reveal user’s shared political ideology [1]. In the case of protein interaction networks, communities can represent groups of proteins that have similar functionality [2]. Since networks that exhibit community structure are common in many disciplines, the last decade has seen a profusion of methods for automatically identifying communities.

Community detection algorithms rely on the topology of the input network to identify meaningful groups of nodes. Unfortunately, real networks are often incomplete and suffer from missing edges. For example, social network users seldom link to their complete set of friends; authors of academic papers are limited in the number of papers they can cite, and can clearly only cite already-published papers. Missing edges can also be a result of the data collection process. For instance, Twitter often limits its data feed to only a 10% “gardenhose” sample: constructing the mention graph from this data would yield a graph with many missing edges [3]. Datasets crawled from social networks with privacy constraints can also lead to missing edges. In the case of protein-protein interaction networks, missing edges result from the noisy experimental process used to measure pairwise interactions of proteins [4]. Community detection algorithms rarely consider missing edges and so even a “perfect” detection algorithm may yield wrong results when it infers communities based on incomplete network information.

One straightforward approach for improving community detection in incomplete networks is to first “repair” the network with *link prediction*, and then apply a community detection method to the repaired network [5]. The link prediction task is to infer “missing” edges that belong to the underlying true graph. A link prediction algorithm examines the incomplete version of the graph and predicts the missing edges. Although link prediction is a well-studied area [6, 7], little attention has been given to how it can be used to enhance community detection. Imputing missing edges using link prediction can result in the addition of both correct intra-community and incorrect inter-community links. If one were to simply run a link predictor and cluster the resulting network, the output can only be improved if the link predictor accurately predicts links that reinforce the true community structure.

**Fig 1:**
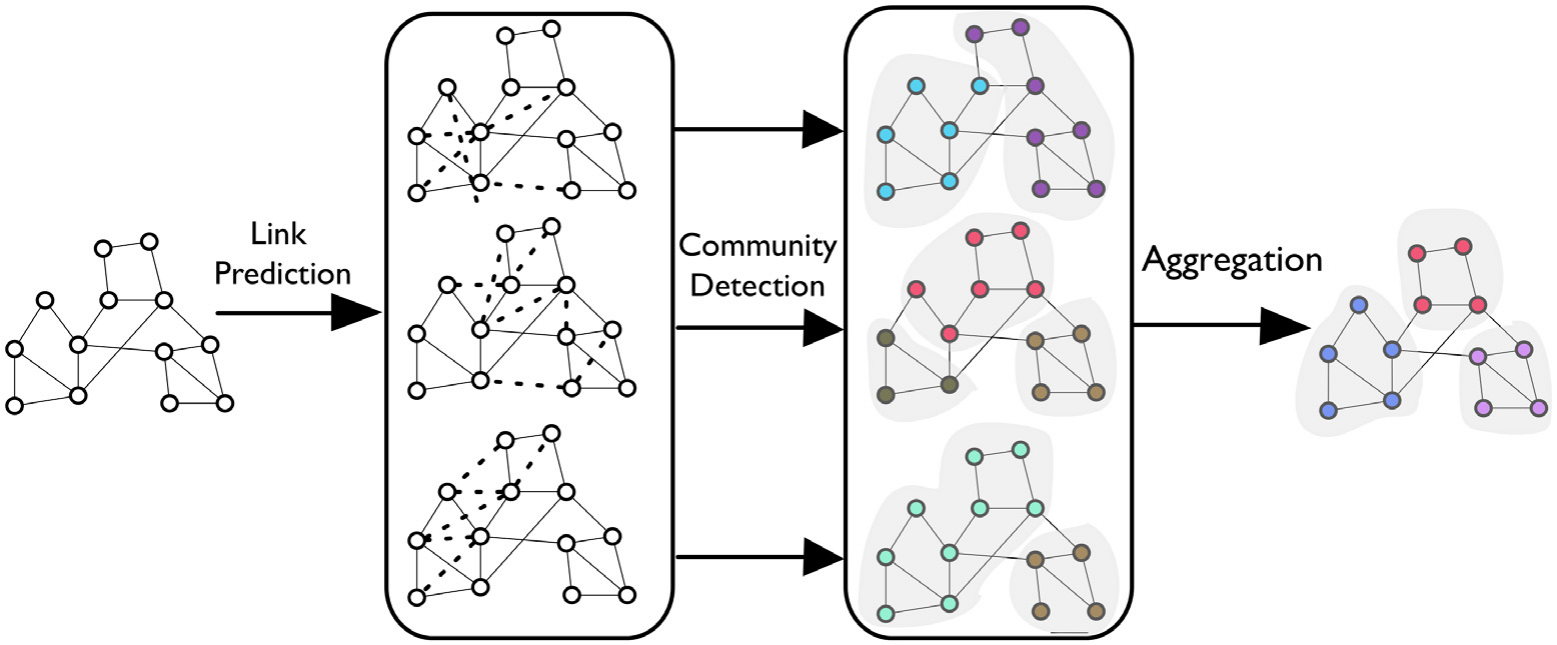
System Diagram. Diagram describing processing steps of EdgeBoost.

We propose the EdgeBoost method (Fig 1), which repeatedly applies a non-deterministic link prediction process, thereby mitigating the inaccuracies in any single link-predictor run. Our method first uses link prediction algorithms to construct a probability distribution over candidate inferred edges, then creates a set of imputed networks by sampling from the constructed distribution. It then applies a community detection algorithm to each imputed network, thereby constructing a set of community partitions. Finally, our technique aggregates the partitions to create a final high-quality community set.

An important and desirable quality of our method is that it is a *meta-algorithm* that does not dictate the choice of specific link prediction or community detection algorithms. Moreover, the user does not have to manually specify any parameters for the algorithm. We propose an easy-to-implement, black-box mechanism that attempts to improve the accuracy of any user-specified community detection algorithm. The open-source implementation of EdgeBoost can be found at https://github.com/mattburg/EdgeBoost

## Related Work

**Community Detection Overview**—There are many variants of the community detection problem: communities can be disjoint, overlapping, or hierarchical. The problem of detecting disjoint communities of nodes is the most popular and what we focus on in this work. While the other variants, especially overlapping community detection, are of growing interest, detecting strict partitions is still a hard and relevant problem. In fact, recent work [8], has shown that disjoint algorithms can perform better than overlapping algorithms on networks with overlapping ground truth. We chose a collection of six algorithms to test our system on: Louvain [9], InfoMap [10], Walk-Trap [11], Label-Propagation [12], Significance [13] and Surprise [14, 15]. We chose Louvain and Infomap for many of our experiments because both performed well in recent comparisons [8, 16, 17]; Infomap is typically superior in quality while Louvain is more scalable.

**Ensemble Community Detection**—Though single-technique community detection is by far the most common, a number of recent projects have proposed ensemble techniques [16, 18, 19]. Aldecoa *et al*. describe an ensemble of partitions generated by different community detection algorithms, which differs from our approach of using the same algorithm and creating the ensemble by creating different networks. Both [18] and [19] present techniques for consolidating partitions generated by repeatedly running the same stochastic community detection algorithm. We implemented both of their methods but neither was suitable for consolidating clusters in our system; this is most likely because the partitions generated from our system have more variation than partitions generated from multiple runs of a stochastic algorithm. At a high-level, our proposed technique is a type of ensemble. Most ensemble solutions take the network as-is and assume that a “vote” between algorithms will produce more correct clusters. While this may work in some situations, bad input will often reduce the performance of all constituent algorithms (possibly in a systematic way) and therefore the overall ensemble. Our proposed method is novel in its iterative application of link prediction to increase the efficacy of community detection algorithms.

**Ensemble Clustering**—Ensemble data clustering (for a survey see [20]), first proposed by Strehl *et al*. [21], involves the consolidation of multiple partitions of the data into a final, hopefully higher quality partitioning. While many of the ensemble clustering methods share a similar work flow to our method, the fact that these techniques were developed for data clustering and not community detection make them distinct from our work. For instance, Dudoit *et al*. use bootstrap samples of the data to generate an ensemble of partitions, which in the case of network community detection would be difficult since networks have an interdependency between nodes, and nodes cannot be sampled with replacement like data in euclidean spaces. Monti *et al*. [22] propose a consensus clustering technique with the goal of determining the most stable partition over various parameter settings of the input algorithm. Similar to our work, many ensemble clustering algorithms [21–24] use a consensus matrix as a data structure to aggregate the ensemble of partitions. Unlike previous methods [21, 23], which use agglomerative clustering to compute the final partition we propose an aggregation algorithm that uses connected components, which is not possible on data clustering problems.

**Community Significance**—In the community detection literature, techniques have been proposed for both evaluating the significance/robustness of communities, as well as, for detecting significant communities. Karrer *et al*. [25] propose a network perturbation algorithm for evaluating the robustness of a given network partition. Methods have also been developed [26, 27] that measure the statistical significance of individual communities. Our goal, however, is not to generate confidence metrics on communities but rather to generate more accurate communities overall. Previous work has also proposed techniques for finding significant communities using sampling based techniques [10, 28, 29]. Rosvall *et al*. and Mirshahvalad *et al*. propose algorithms for detecting significant communities by clustering bootstrap sample networks and identifying communities that occur consistently amongst the sample networks. The method proposed by Gfeller *et al*. attempts to identify significant communities by finding unstable nodes using a method based on sampling edge weights. Their methods differ from ours in that they create samples from the *existing* network topology. Most similar to our work is the paper by Mirshahvalad *et al*. [5] which attempts to solve the problem of identifying communities in sparse networks by adding edges that complete triangles. Their method is simply to add a fixed percentage of triangle completing edges and cluster the resulting network; in contrast, our approach involves the repeated application of any link prediction algorithm.

**Community Granularity**—The problem of detecting communities at various levels of granularity is a well studied and related problem. Work by [30, 31] has analyzed the “resolution limit” of detecting communities at all granularities. In response to this resolution problem, many methods [32–35] have been proposed for community detection at different granularities. New objective functions that improve the resolution limit [34] as well as tunable objectives [32, 35] that allow community detection at various resolutions have been proposed. Delvenne *et al*. propose a method for identifying the stability of communities by using the Markov time of a random walk on the network. Granularity is a related problem in that missing edges can lead to communities detected at wrong granularities. However, these methods do not address the problem of detecting communities on incomplete networks.

## Problem Formulation

### Communities in Incomplete Networks

To motivate the need for algorithms that are robust to missing edges, we experimented on existing community detection algorithms. To test these algorithm’s sensitivity to missing edges on a range of networks, we utilize the LFR benchmark [36]. LFR creates random networks with *planted partitions* (i.e., ground-truth community structure), parametrized by: number of nodes, mixing parameter *μ*, and exponent of degree and community size distributions (see [36] for a full description). The mixing parameter is a ratio that ranges from only intra-community edges (0) to only inter-community edges (1). Previous studies [16, 17] have compared the quality of community detection algorithms using the benchmarks and used *μ* as the variable parameter, roughly capturing how difficult a network is to cluster. As we are concerned with characterizing the effect of missing edges, we modify the LFR benchmark by randomly deleting edges from the networks it generates. We denote the parameter *δ* as the percentage of removed edges.

The goal of our analysis is to characterize the effect of both *μ* and *δ* on two metrics: Normalized Mutual Information (NMI) and the Relative Error (RE) of the size of the inferred partitions. NMI is a standard information theoretic measure for comparing the planted partition provided by the benchmark to the inferred partition produced by the algorithm. There are various metrics classified as normalized mutual information; the metric we use throughout this paper is the normalization of mutual information (I) based on maximum entropy (H) of the two partitions.

$$\begin{array}{c} NMI\left(U,V\right)=\frac{I(U,V)}{max(H(U),H(V))} \end{array}$$ (1)

We define RE as the relative error of the number of communities inferred by the algorithm *C* compared to the number of communities C* in the planted partition:

$$\begin{array}{c} RE:=\frac{C-C*}{C*} \end{array}$$ (2)

**Fig 2:**
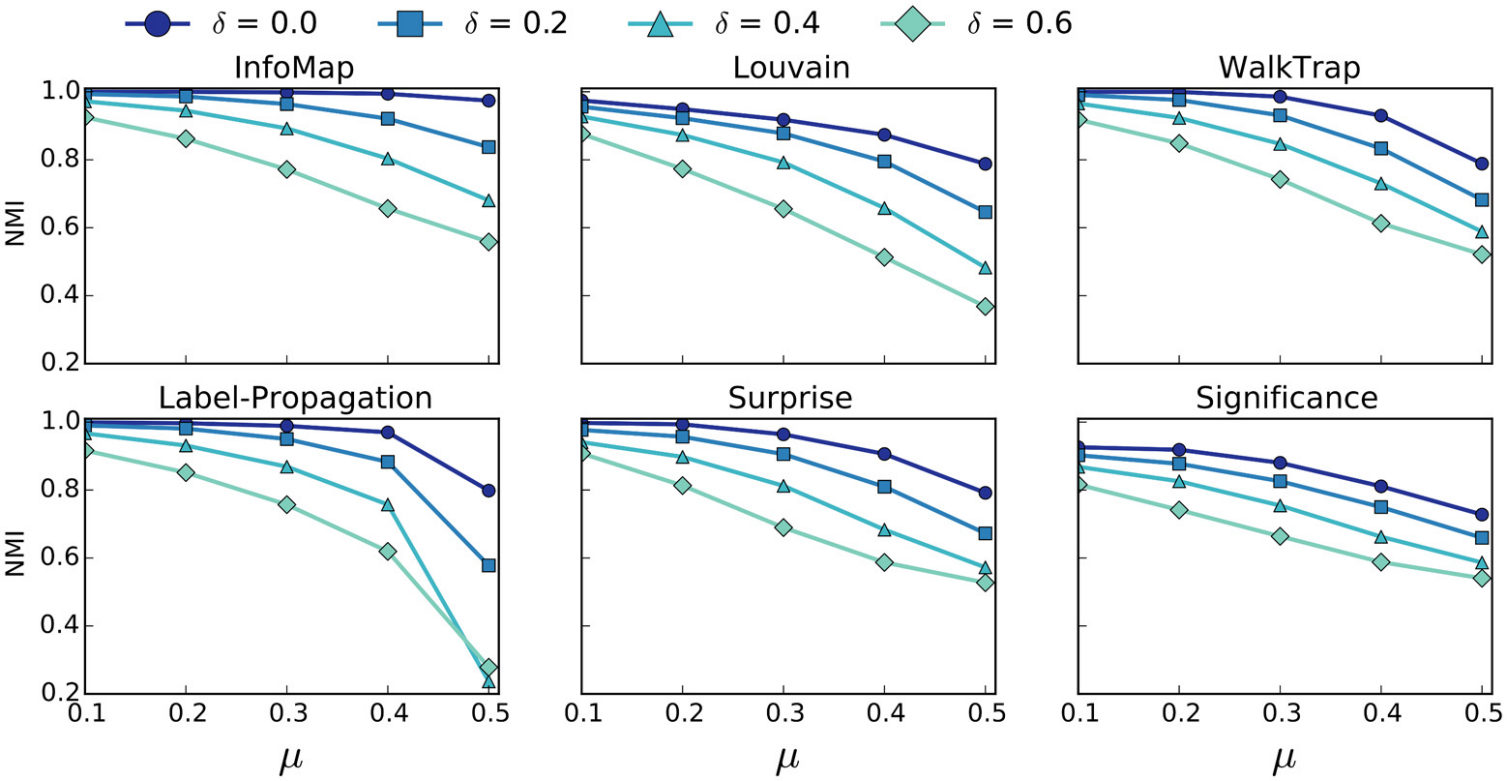
NMI of Baseline Community Detection Methods. NMI of six community detection algorithms with varying percentages of removed edges *δ*. Error bars are not included because standard error is too small.

**Fig 3:**
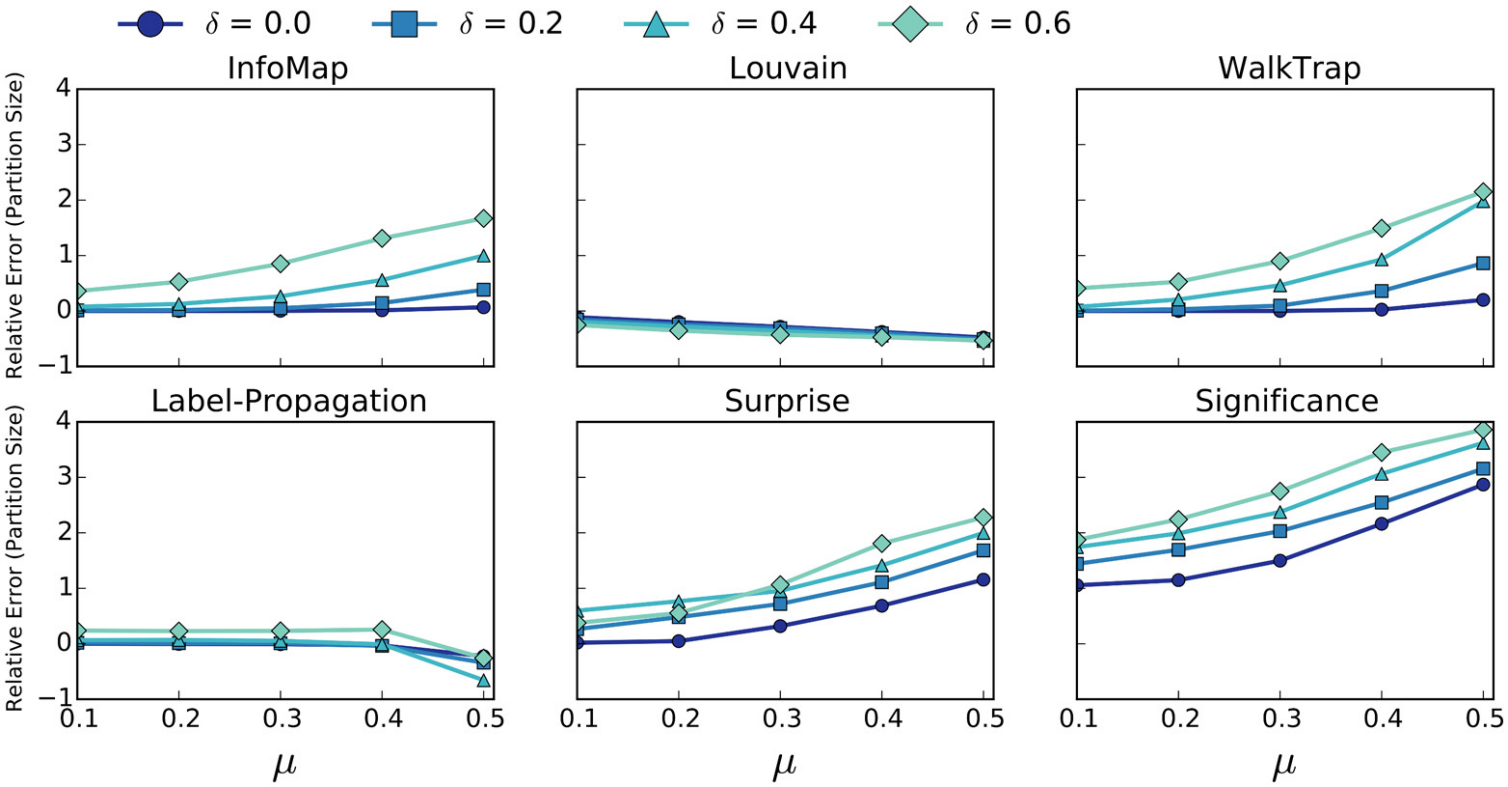
RE of Baseline Community Detection Methods. RE of six community detection algorithms with varying percentages of removed edges *δ*. Error bars are not included because standard error is too small.

Since NMI can decrease for a variety of reasons (shifted nodes, shattered or merged communities), we include RE as a means to determine the more specific effects that missing edges can have on community detection. Each point in Figs 2 and 3 are generated by averaging the corresponding statistic over 50 random networks generated by our modified LFR benchmark. We set static values for the following benchmark parameters: Number of nodes (1000), the average degree (10), the maximum degree (50), the exponent of the degree distribution (-2), exponent of the community size distribution (-1), minimum community size (10), and maximum community size (50). We varied parameters, such as “number of nodes” and “average degree”, finding qualitatively similar results for the effect of *δ* on NMI and RE. Similar to previous research [5], we select an “average degree,” that results in the sparse networks that motivate the need for the methods presented in this paper.

Fig 2 shows how NMI varies with respect to *δ* and *μ* for six popular community detection algorithms. We limit the values of *μ* to be in the range [0.1, 0.5] becauase it has been shown that LFR networks with *μ* values of 0.5 and higher do not reflect the expected properties of real world networks [37]. All of the algorithms behave in a qualitatively similar manner: as *δ* increases, the NMI score decreases. Similar to previous studies, InfoMap scores best with respect to *μ* and not surprisingly is also the most robust to missing edges. More interesting are the results in Fig 3 which show how the number of inferred communities differs with respect to the number of communities in the planted partition. Four of the six algorithms show a trend of detecting too many communities both as a function of *μ* and *δ*, while only the Louvain and Label-Propagation algorithms detect fewer than the correct number of communities on average. Modularity is known to suffer from a resolution limit [30], meaning that the measure tends to favor larger communities. Since Louvain uses modularity as its objective function, it is not surprising that Louvain, on average, infers communities that are larger than in the planted partition. Overall, it is more often the case that missing edges will cause community detection algorithms to “shatter” ground truth communities, sometimes producing 2-3 times more communities. Both in terms of NMI and RE, all 6 algorithms show a significant deterioration in community detection quality, once again, underscoring the need for algorithms that are robust to missing edges. We have also included heat map versions of the Figs 2 and 3 in the supporting information section, labeled as S1 and S2 Figs respectively.

### Link Prediction for Enhancing Community Detection

The ideal scenario for community detection is one where a network consists of only intra-community edges and where the detection of communities reduces to the problem of identifying weakly connected components. The reality is that we rarely find such clean graphs as edges can be “missing” for anything ranging from sampling to semantics. This last factor is important as a missing edge between nodes in the same community is not necessarily incorrect—the semantics of a network does not necessitate an explicit relationship between users in the same community. In the case of an ego-network on Facebook, for example, not all friends in the same community actually know each other as they may be grouped because they attend the same college as the ego-user. Similarly, a biological network may have a set of proteins working in concert as part of a functional “community” but many do not form a clique as the edges represent (up or down)-regulation. In both scenarios the edges that are missing are implicit edges representing the intra-community links (e.g., an edge representing the relationship *in-the-same-community-as*). These intra-community links can be thought of as analogous to strong ties as proposed by Granovetter [38]. It is these intra-community edges, whether they are implicit or explicit, that can have severe impact on the detection of communities.

The hypothesis of this paper is that by recovering edges in incomplete networks, community detection quality can be improved. If link prediction is to be an effective strategy at recovering lost community structure, it must be accurate at predicting intra-community edges that reinforce communities. If the link prediction algorithm has too high a false-positive rate, thereby predicting too many inter-community links, it is likely to degrade community detection performance. Using the modified LFR benchmark, we analyzed the intra-community precision of various link prediction algorithms over a range of *μ* and *δ* values. We do not intend to exhaustively test all of the link prediction algorithms proposed in the literature, but we select three computationally efficient techniques that are among the best [6, 7]: Adamic-Adar (AA), Common-neighbors (CN), and Jaccard.

**Fig 4:**
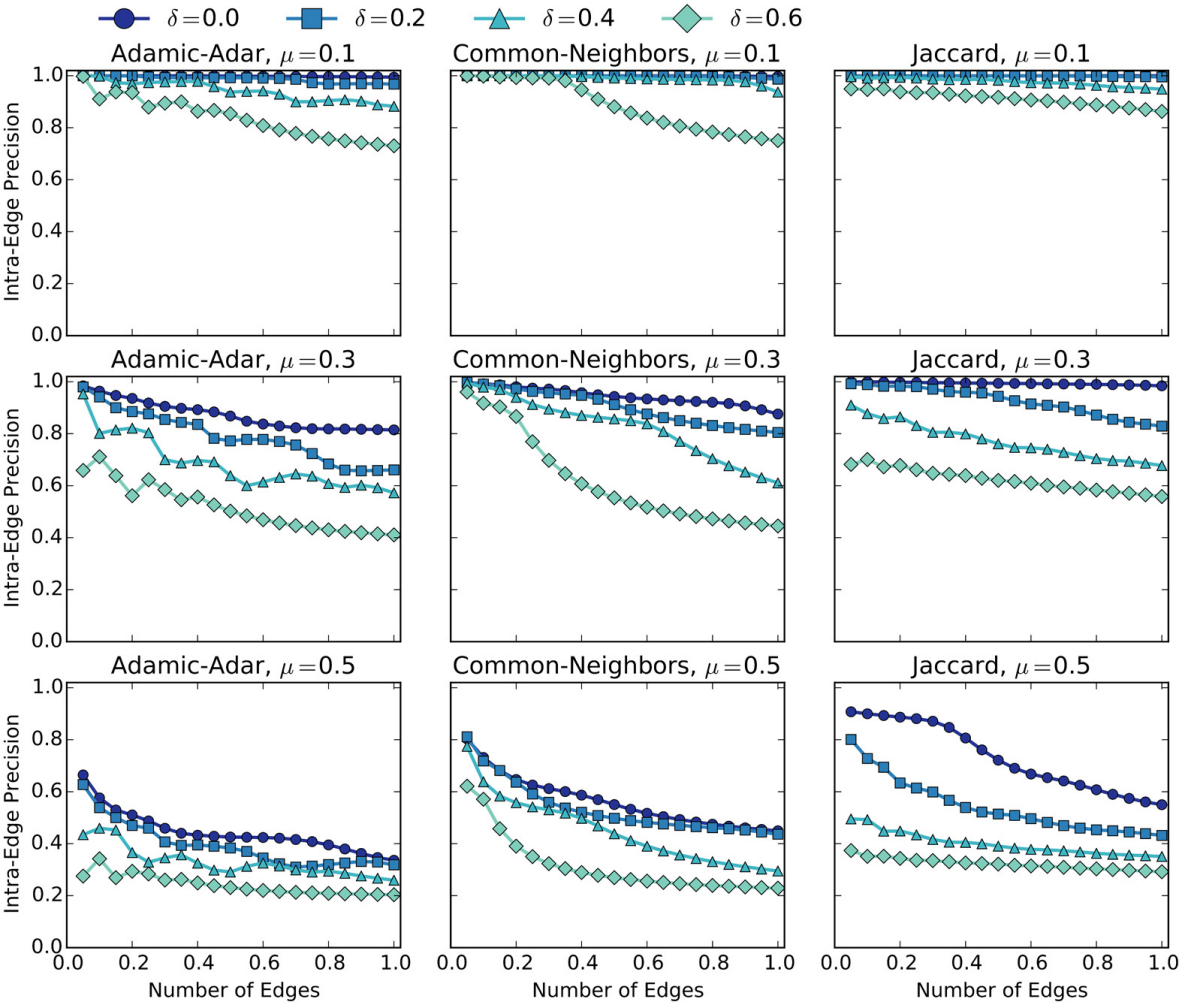
Intra-edge Precision of Link Prediction. Precision plots of three link prediction algorithms: Adamic-Adar (left), Common Neighbors (middle), and Jaccard (Right) for various values of mixing parameter *μ*: 0.1 (top), 0.3 (middle), and 0.5 (bottom). The *X*-axis corresponds to number of top-*k* edges as scored by the link prediction algorithm as a percentage of the number of edges in the network. Intra-edge precision is on the *y*-axis.

Each of these algorithms can produce a score for missing edges that complete triangles in the input network, allowing us to create a partial ordering over the set of missing edges. Fig 4 shows the results from our experiment. For each plot, the *y*-axis represents the intra-edge *precision*-*at*-*k* metric, which is the percentage of intra-edges in the top-*k* edges of the ranking. The *x*-axis represents the edge-percent value, which is the number of top-*k* edges as a percentage of the total number of edges in the original network (before random deletion). For example, if the original network had 2000 edges, then an edge-percent value of 20% would correspond to selecting the top-400 edges and a intra-edge precision value of 80% would correspond to 320 of those edges being intra-community edges. By varying *k* we are able to observe the classification quality inferred by the ranking produced by each link-predictor.

In Fig 4 we first notice that as with community detection, link prediction performance decreased as a function of both *δ* and *μ* value. For low *μ*, all link prediction algorithms are capable of achieving high intra-edge precision even for *δ* values of 60%, but the quality of link prediction drops significantly for high levels of *μ*. For *μ* above 0.5, any link-predictor that uses the number of common-neighbors as a signal will do poorly, since the majority of a node’s neighbors belong to different communities. The Jaccard algorithm maintains the highest level of precision as a function of the number of edges. While the AA algorithm sometimes outperforms Jaccard, AA is only better for low values of *k*.

The results in Fig 4 show that link prediction can be effective at imputing intra-community edges, especially for sparse networks that have lower *μ* values. The results also show that for networks with high *μ* and *δ* values, the top-scoring edges as predicted by all three link prediction algorithms contain a large percentage of inter-community links. While this demonstrates the feasibility of using link prediction to recover missing intra-community edges, we do not know how to set the parameters (e.g., the *k* value to use for partitioning the ranked edges) for real-world networks. We will return to this, but first we formalize the problem.

Let *G* = (*V*, *E*) be the input network, and the set *E*_missing_ = (*V* × *V*)\*E* denote the set of missing edges in *G*. We formally define a link-predictor L as a function that takes any pair of nodes (*x*, *y*) in *E*_missing_ and maps them to a real number.

$$\begin{array}{c} ℒ:E_{\text{missing}}\;\mapsto \;R \end{array}$$ (3)

A community detection algorithm can be formally described as a function *C* that takes as input any network *G* and produces a disjoint partition of the nodes {*C*_1_, *C*_2_,…,*C*_*k*_}.

The most naïve algorithm for enhancing community detection consists of a few simple steps. First, score missing edges in *G* using L. Next, select the top-*k* missing edges according to the link-predictor and add these edges to *G*. Lastly, apply the algorithm *C* to the imputed network. However, simply adding links with high scores for networks with large *μ* and *δ* values may be problematic, since many of these links can be inter-community, thereby having a negative effect on community detection.

**Fig 5:**
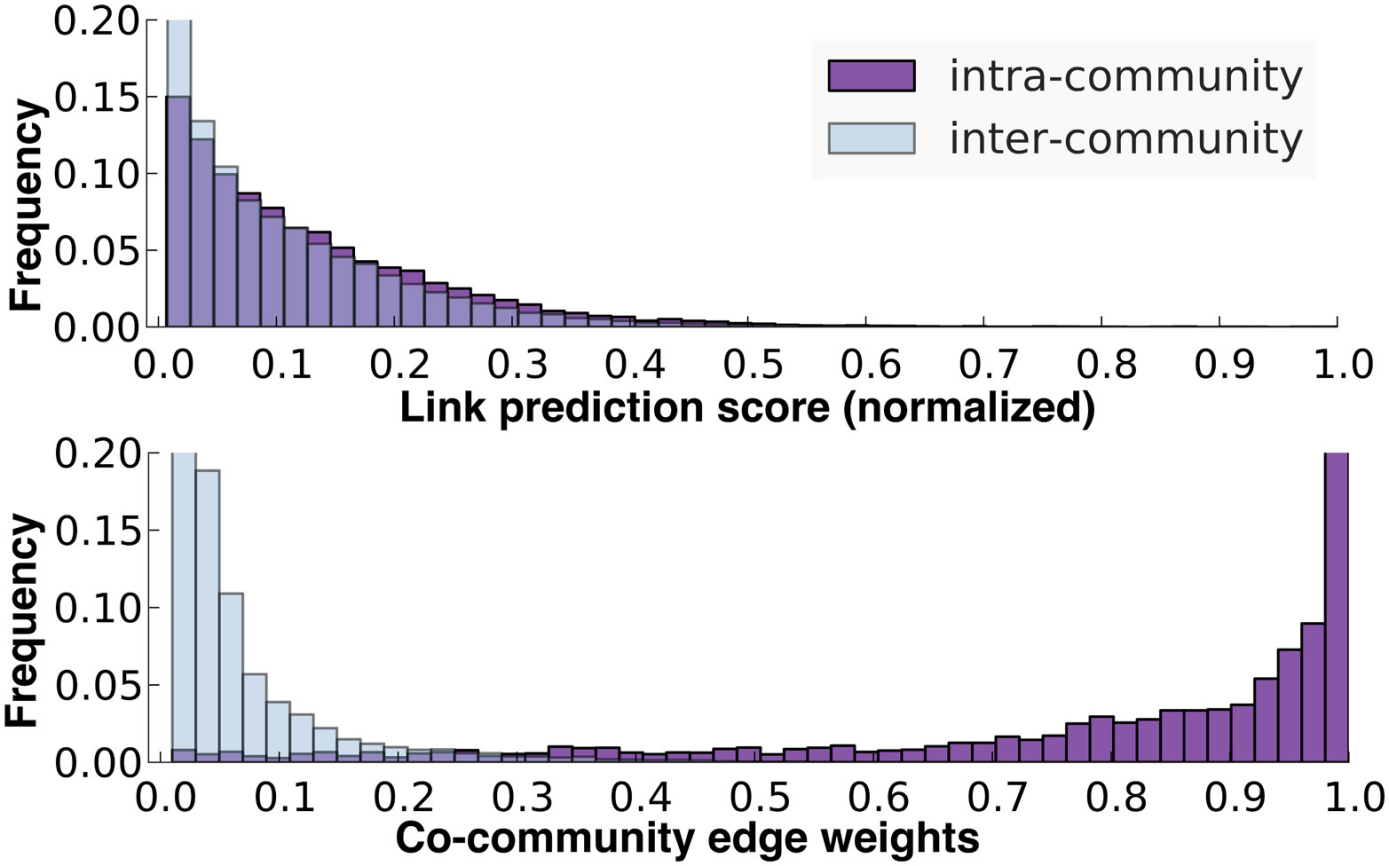
Edge Weight Distributions. Histogram of edge weights on a benchmark graph with *μ* = 0.4 and 20% of the edges removed: scores from AA link predictor (top) and weights of co-community network (bottom).

An intuition for why this naïve algorithm does not work is illustrated in the top histogram of Fig 5. The plot shows the score distribution of both intra- and inter-community edges predicted by the *AA* link predictor on a randomly generated benchmark network. The distributions of the intra-community edges substantially overlaps with the inter-community distribution, thereby making any choice of a threshold for adding links not helpful for community detection. In addition, as this plot shows, the top-*k* edges only comprise of a small percentage of the total set of intra-community edges. By simply selecting from the top-*k* scoring edges, many of intra-community edges that are lower ranked will never be selected. As demonstrated in Fig 4, the choice of *k* can have a significant impact on the quality of the edges, therefore selecting the right *k* becomes a challenge when the complexity and sparsity of the network is unknown.

## Methods

We contacted the original authors of the Facebook dataset (https://snap.stanford.edu/data/egonets-Facebook.html) used in our experiments. This dataset was collected in accordance with Facebook terms of service and with oversight from an IRB.

Our core observation is that link prediction in both high-*δ* and high-*μ* settings is brittle: it can carry information, but for a single prediction is likely to be wrong. Therefore, we propose an improved method for applying link prediction to enhance community detection.

**Procedure 1 EdgeBoost Algorithm**

**Input:** A network *G* = (*V*, *E*), link-predictor L, community detection algorithm *C*, number of iterations *n*

**Output:** A partition P* of the vertices in *G*

 1: $E_{\text{missing}}=L\left(G\right)\;$                     ⊳ score edges in *G*

 2: D = Imputation(*E*_missing_)          ⊳ create edge distribution

 3: *P* = []                 ⊳ initialize list of partitions

 4: **for***i* ← 1, *n***do**

 5:  *k* ∼ *U*(1, |*E*|)

 6:  $\left\{e_{1},e_{2},\ldots ,e_{k}\right\}∼D$                   ⊳ sample *k* edges

 7:  *G*_*i*_ = (*V*, *E*∪{*e*_1_,…,*e*_*k*_})                   ⊳ impute *G*_*i*_

 8:  $p_{i}=C\left(G_{i}\right)$                          ⊳ cluster *G*_*i*_

 9:  *P* = *P* ∪ *p*_*i*_

10: **end for**

11: P* = AggregationFunction(*G*, *P*)

12: **return**P*

In order to mitigate the potential side effects of imperfect link prediction, we propose a sampling based algorithm that repeatedly applies link prediction to the input network. The EdgeBoost pseudo code is shown in Algorithm 1 and proceeds in four steps. First, it uses a link prediction function to score missing edges and constructs a probability distribution over the set of missing edges (lines 2-3). The algorithm repeatedly samples a set of edges from this probability distribution, adding these sampled edges to the original network, and runs community detection on the enhanced network (lines 5-7). Each iteration produces a new set of communities which are added to the set of partitions (lines 8-9). After the sample-detect-partition sequence is executed many times, we aggregate the overall set of observed partitions (line 11) to produce a final clustering.

### Network Imputation

The network imputation component of EdgeBoost uses the input network and a link prediction algorithm to produce a probability distribution over the set of missing edges. The number of edges sampled during each iteration of the imputation procedure (lines 5-6) is a uniform random number between 1 and the size of the input network. We experimented with many values of *k* and found this to work as well as when *k* was fixed.

We propose an imputation algorithm that constructs a distribution in which the probability of drawing an edge corresponds to its score produced by the link predictor. Missing edges that are scored higher by the link-predictor will have more probability mass than lower scoring edges. The probability function constructed from this process is:

$$\begin{array}{c} P\left(X=x\right)=\frac{ℒ(x)}{\sum_{y\in E_{\text{missing}}}ℒ\left(y\right)} \end{array}$$ (4)

Our imputation algorithm is more likely to pick higher scoring edges, which can result in a fairly accurate selection of intra-community edges as shown in the *Link-Enhanced Community Detection* section. At the same time, even low scoring edges have probability mass, which is important since for some networks, intra-community edges can also be low scoring.

### Partition Aggregation

Having generated many possible “images” of our original graph via network imputation, we can apply community detection algorithms to each. Each execution of the algorithm produces a partition—possibly unique—based on the input graph. After generating many such partitions, we use *partition aggregation* to produce a final output. Previous ensemble clustering techniques [18, 19], construct a *n* × *n* consensus matrix that represents the co-occurrence of nodes within the same community. The goal of such a data structure is to summarize the information produced by the various partitions. We propose a similar data structure, a co-community network *G*_cc_, which consists of nodes from the input network and edges with weights that correspond to the normalized frequency of the number of times the two nodes appear in the same community.

The *G*_cc_ graph is a transformation of the input network into one that represents the pairwise community relationships between nodes, rather than the functional relationships defined by the semantics of the input network *G*. *G*_cc_ links nodes that appear in the same community, and weights them based on frequency or co-occurence (i.e., the edge between two nodes has a normalized weight equal to number of times the two nodes appear together in the same community over all partitions). Thus, *G*_cc_ exhibits community structure representing communities that appeared frequently in the input partitions. As shown in the lower plot of Fig 5, there is a clear distinction between the intra-community and inter-community edge-weight distributions in *G*_*cc*_. A simple mechanism for identifying a final “partitioning” is to remove all edges for which we have low confidence (i.e., inter-community edges) and study the resulting connected-components (CC). We parameterize the pruning with a threshold *τ* and prune edges below that value. The semantics of the resulting graph is that all pairs of linked nodes have been seen in the same community at least *τ* percentage of times and consequently all nodes captured in a CC maintain this guarantee.

**Fig 6:**
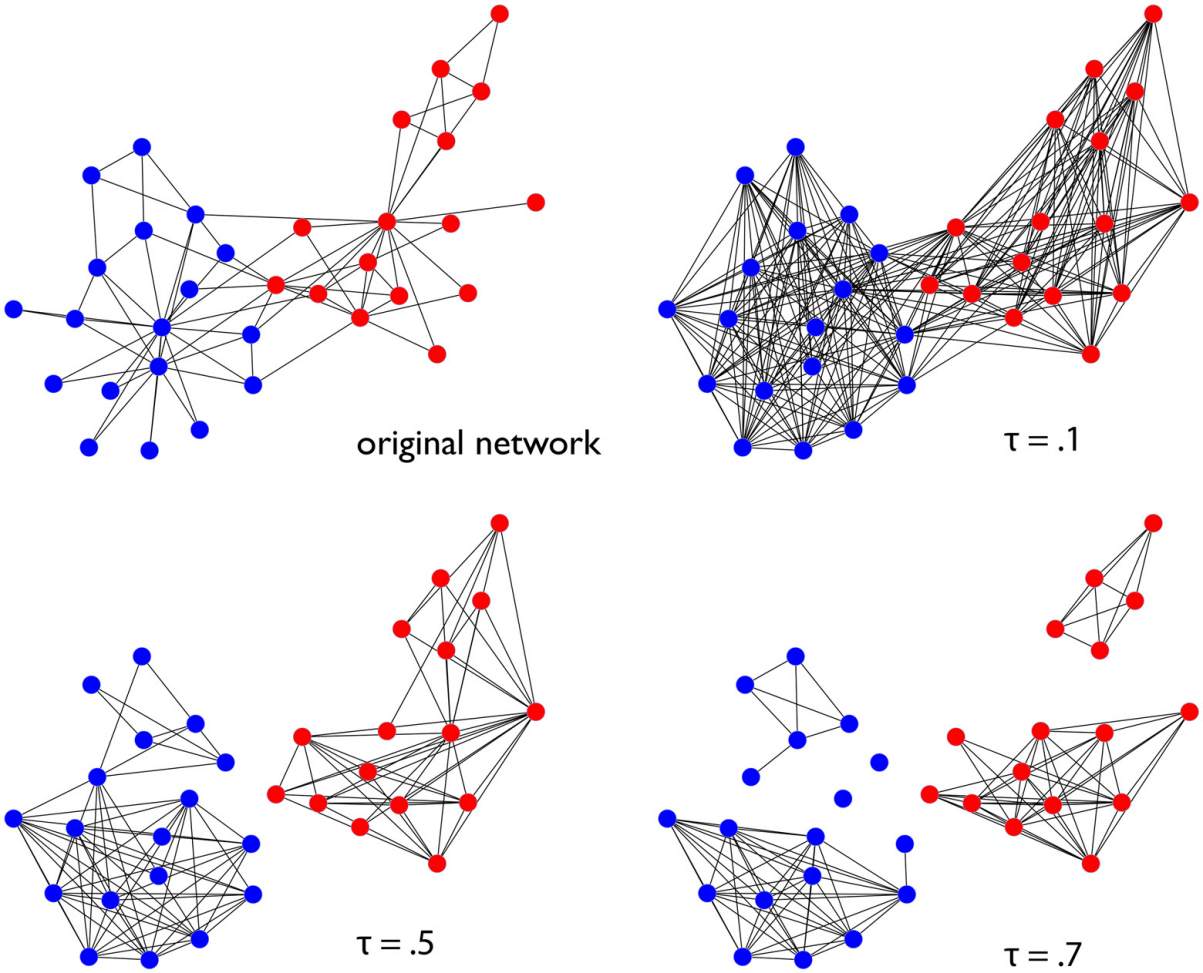
Karate Club Co-community Network. Visualization of the co-community network for “Zachary’s karate club” network. Each panel shows the network pruned at various thresholds *τ*.

Fig 6 shows an example of a co-community network pruned at various thresholds. The network in this diagram is the famous *Z*achary’s karate club [39], the colors of the nodes denote the ground-truth community assignments of each node. The original network is shown in the upper left quadrant, and the remaining quadrants show the co-community network pruned at different thresholds. As we can see in the upper right quadrant, if we threshold at a small value of *τ* we are almost certain to obtain a network with one large connected component. This is due to the fact that given enough iterations of the link prediction/community detection loop we are likely to find at least a few cases where nodes that would ordinarily fall into two communities are placed into the same one. At *τ* = 0.5 we see the CC’s reflect the community structure in the original network (the “correct partition”). As we increase the threshold the two true communities are further shattered into sub-communities, leaving some nodes completely isolated. One can interpret the connected components at these higher levels of *τ* as capturing the *core members* of the true communities: members who co-occur with each other a very high percentage of time and do not co-occur often with nodes outside of their community.

While *τ* may be set manually—appropriate for some applications when some level of confidence is desirable—there are other applications where we would prefer that this threshold be chosen automatically. As the last module of our framework we propose a way for selecting a *τ* and constructing a final partitioning given that chosen value. Since the edge weights in *G*_*cc*_ correspond to the fraction of times two nodes appear in the same community, they are rational numbers. We can therefore enumerate all the possible values of *τ*, $\left\{\frac{1}{n},\frac{2}{n},...,\frac{n}{n}\right\}$ on the interval [0, 1]. At each value of *τ* we prune all edges with weights less than *τ* and compute the partition of *G*_cc_ that corresponds to the connected-components. We then score this partition according to Eq 6 and select the threshold and corresponding partition that maximizes this score. Our algorithm for automatically choosing *τ* does add computational overhead as compared to simply selecting a *τ* manually. We evaluate the selection of a fixed *τ* threshold for the LFR networks in the experiments section.

In previous work, Monti *et al*. [22] propose a formula for computing the “consensus” score of an individual cluster. For a given community *C*_*k*_, that is of size *N*_*k*_, their score sums the co-community weights and divides it by the maximum possible weight. *G*_*cc*_(*i*, *j*) corresponds to the fraction of times nodes *i*, *j* were grouped together in the same community.

$$m_{k}=\frac{1}{\left(\begin{array}{c} N_{k}\\ 2 \end{array}\right)}\underset{\begin{array}{l} i,j\in C_{k}\\ \;i<j \end{array}}{\sum }G_{cc}\left(i,j\right)$$ (5)

We score a partition *p*_*τ*_ parameterized by a threshold *τ* by taking the weighted sum of the scores *m*_*k*_ for each community in the partition. We use a weighted sum because the score contribution of each community should be commensurate with its size.

$$\begin{array}{c} S\left(p_{\tau }\right)=\frac{N_{k}}{N}\sum_{k\in p_{\tau }}m_{k} \end{array}$$ (6)

If the final partition has any singleton nodes that do not belong to any community we connect each stray node to the community to which it has the highest mean edge weight to in the un-pruned co-community network.

## Experiments

We have conducted a series of experiments to test EdgeBoost on the LFR benchmark networks, standard real-world networks (e.g karate club), and a set of ego networks from Facebook. First, we present a comparison of EdgeBoost with different community detection methods. Subsequent experiments include an analysis of various parameter settings of EdgeBoost. The Facebook dataset used in our experiments was collected in accordance with Facebook terms of service and with oversight from an IRB.

### Comparing EdgeBoost with Different Community Detection Methods

**Fig 7:**
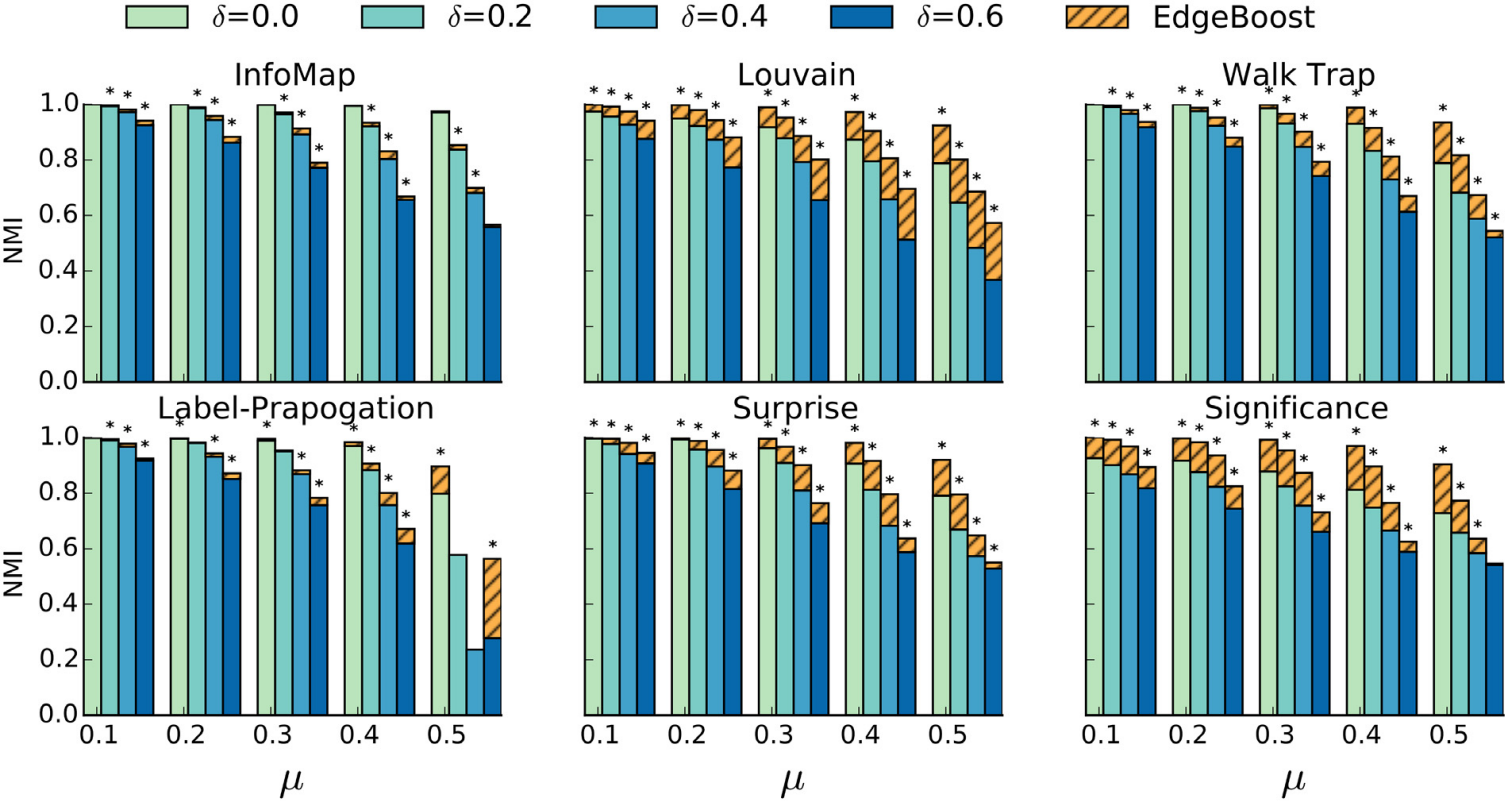
EdgeBoost Performance on LFR Networks. Performance of six popular community detection algorithms on the LFR benchmark networks. Dashed yellow bar shows the improvement of EdgeBoost over using the baseline community detection method.

Similarly to the analysis we performed in the *Communities in Incomplete Networks* section, we evaluate our methods against the LFR benchmark over various settings of the mixing ratio *μ* and the percentage of missing edges, *δ*. In Fig 7 we show the performance gain (striped yellow bars) of EdgeBoost for six different community detection algorithms: InfoMap, Louvian, WalkTrap, Label-Propagation, Surprise, and Significance. The number of imputation iterations is fixed at 50 for both algorithms and the bars are generated by averaging over 50 randomly generated networks. While not shown in Fig 7, we tested all 3 link prediction algorithms and did not find a substantial difference. Keeping with our link prediction analysis in the *Link Prediction for Enhancing Community Detection* section, Jaccard slightly outperformed the other methods, so we chose Jaccard as the link prediction algorithm for EdgeBoost. We ran a Mann-Whitney U test for each parameter configuration and found that 90% of the results in Fig 7 have a p-value less than 0.05.

We can see from Fig 7 that our method improves performance for almost all input community detection algorithms. One notable exception is that EdgeBoost does not show an increase in performance for the InfoMap algorithm. Our hypothesis is that the objective function that InfoMap uses—which is based off of random walks—does not benefit as much from the imputation of triangle completing edges. Since we show that EdgeBoost can improve the performance of InfoMap on real network data (described in the next section), there may also be a systematic biased produced by the benchmark networks that make improving InfoMap harder. Another exception is that EdgeBoost shows a decrease in performance for the Label-Propagation algorithms at a *μ* value of 0.5 for 2 of the *δ* configurations. As in other studies [19], the Label-Propagation algorithm’s performance becomes erratic at *μ* values of 0.5 or greater, most likely due to the fact that Label-Propagation assumes that a node’s label should be chosen based on the labels of its neighbors. While EdgeBoost is designed to work on stochastic algorithms, and variations of the input network, if an algorithm has too much variation, as is the case with Label-Propagation, it can lead to unpredictable performance.

**Fig 8:**
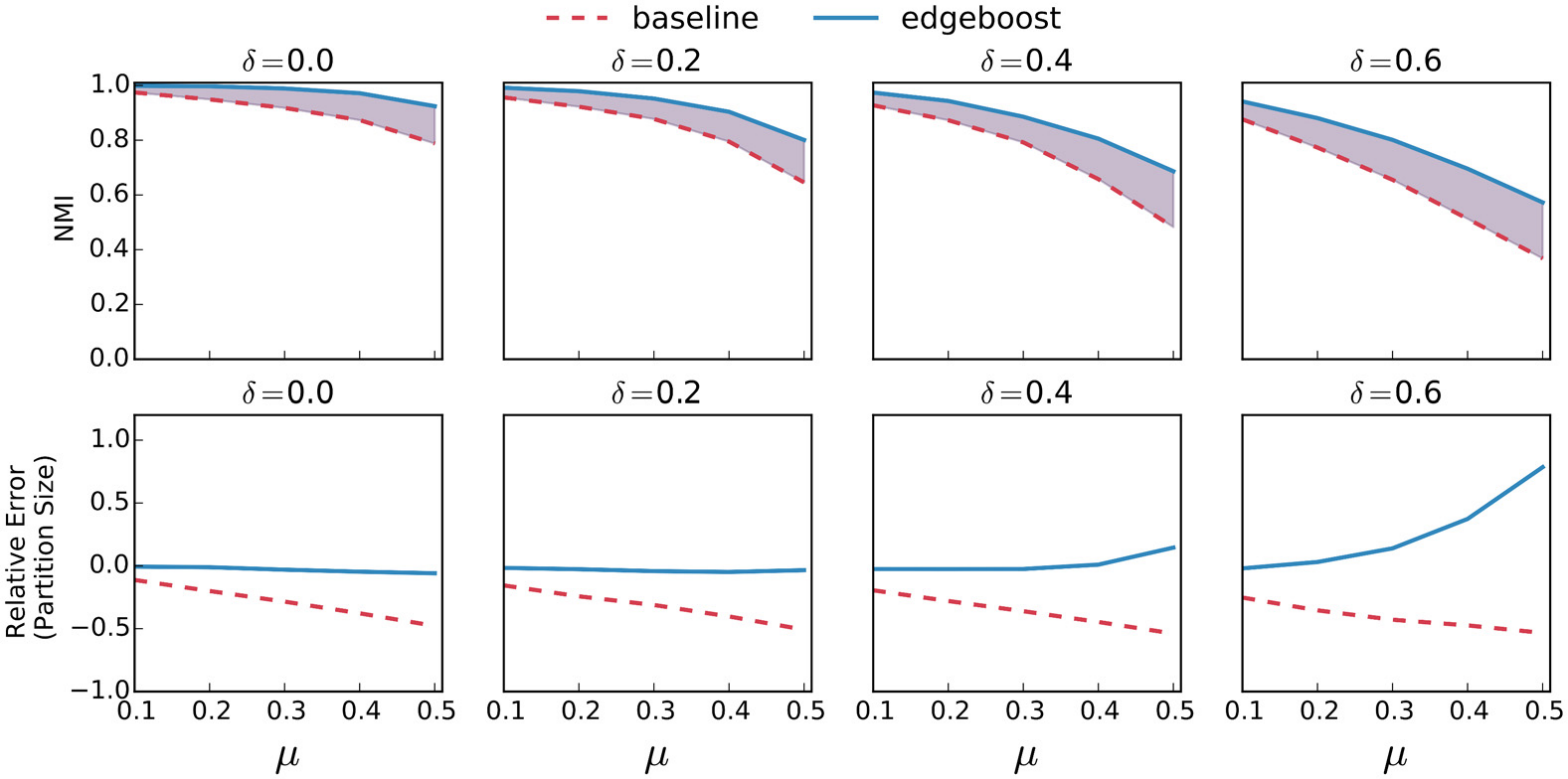
EdgeBoost Paired With Lovain. Performance of EdgeBoost (solid) and the baseline Louvain algorithm (dashed) on LFR benchmarks. The purple shaded region shows the improvement of EdgeBoost for NMI. The bottom row shows the relative error of the partition size.

Fig 8 shows the performance gain of EdgeBoost on the Louvain algorithm in more detail. As our previous analysis showed, the baseline Louvain algorithm tends to detect bigger communities on average than in the planted partition. The bottom row shows that for moderate values of *δ*, EdgeBoost is able to recover the smaller communities in the planted partition. At very high values of *δ* (> = 0.4) a network may be so sparse that the perfect recovery of correct communities is most likely not possible. Even for these high *δ* values, EdgeBoost still shows an improvement in NMI over the baseline method. The Louvain algorithm shows similar performance gains as a function of the *δ* parameter but as seen in Fig 7, other algorithms show more variation with respect to *δ*. We have also included plots that characterize the performance of all the other algorithms in S4, S5, S6, S7 and S8 Figs respectively.

**Fig 9:**
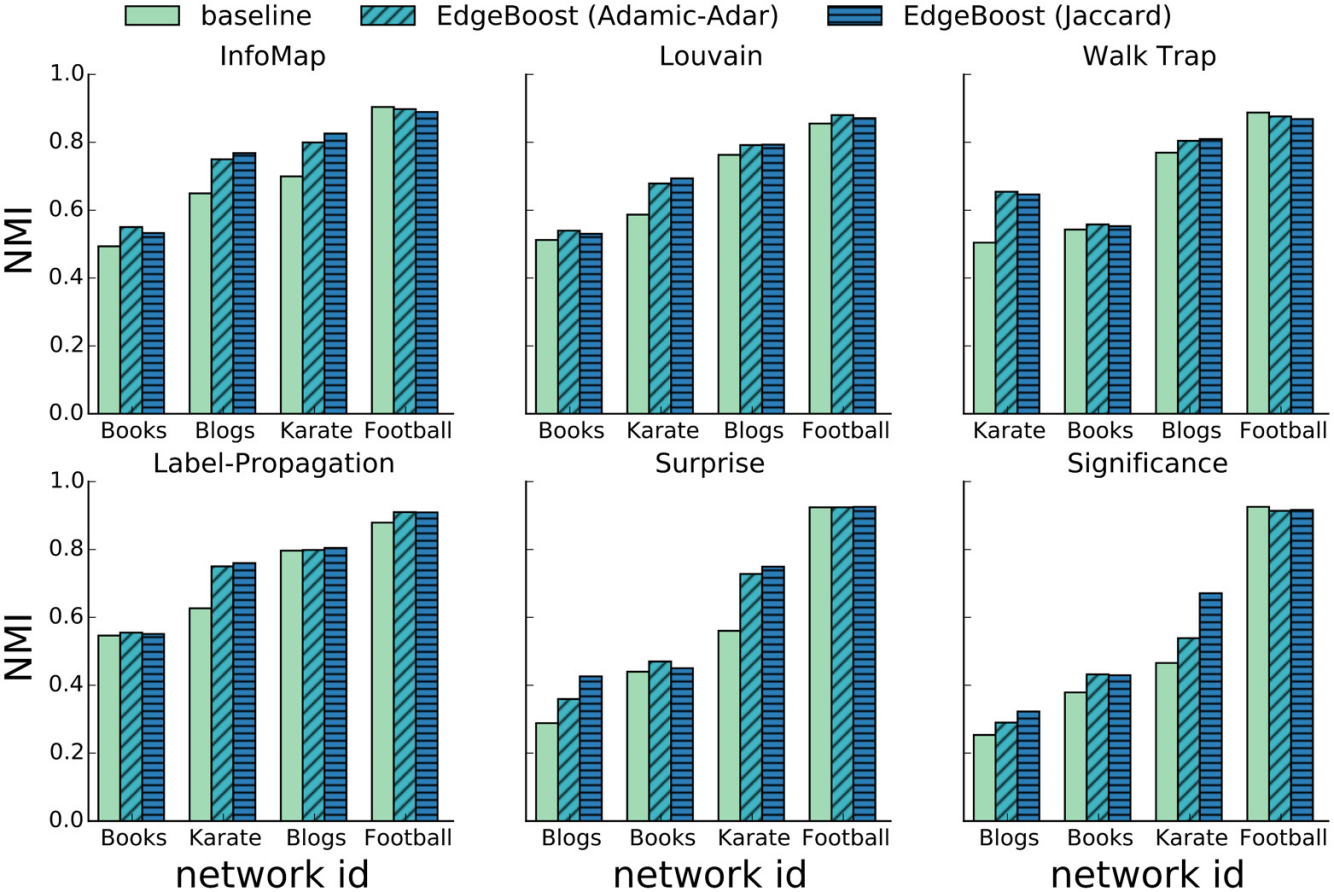
Performance of EdgeBoost on Standard Network Datasets. Comparison of EdgeBoost on set of standard real network benchmarks community detection.

The LFR benchmark captures certain network properties, but it is an imperfect model of real-world networks. To test EdgeBoost on real network data, we also performed experiments on two additional data sets. The first data set consists of a suite of standard networks for benchmarking community detection. The data set includes: Zachary’s *Karate Club* network (Karate) [39], network of political books (Books) [40], blog network (Blogs) [41] and the American college football network (Football) [42]. All of these networks have a ground truth partition such that we can use NMI to evaluate the performance of community detection. Fig 9 shows the results of EdgeBoost on each of the four networks with the same six input community detection algorithms used in the experiment above. In all but three of the 24 algorithm/network configurations, EdgeBoost improves performance by an average of 14%. On the Football network, EdgeBoost does worse with the InfoMap, Label-Propagation, and WalkTrap algorithms, but decreases performance by only an average of 1.6%. Overall, these datasets give some assurance that EdgeBoost can improve performance on real networks.

We also tested EdgeBoost on a data set of Facebook ego-networks [43] that capture all neighbors (and their connections) centered on a particular user. The data set described in the original paper by McAuley *et al*., consists of networks from three major social networks: Facebook, Google+ and Twitter. The Facebook data set is likely the highest quality of the three; it contains ground-truth which was obtained from a user survey that had the ego users for each network provide community labels. The ground truth for the ego-networks from Twitter and Google+ is lower quality since it was obtained by crawling the publicly available lists created by the ego user. As such, for many of the networks, the ground truth consisted of only a small fraction of nodes in the network and for many networks the ground truth consisted of lists with very few members. Since the target of this paper is non-overlapping and complete clustering, we chose to not use the Twitter and Google+ networks due to the sparsity of their ground-truth. The Facebook networks have complete ground-truth labeling, so we used those for evaluation. Despite the Facebook networks being the highest quality of the three datasets, it still contained ground-truth communities of 1-2 users. We pre-processed each network by removing all ground-truth communities with fewer than three nodes. S3 Fig contains a plot of the distribution of community sizes for the Facebook networks.

**Fig 10:**
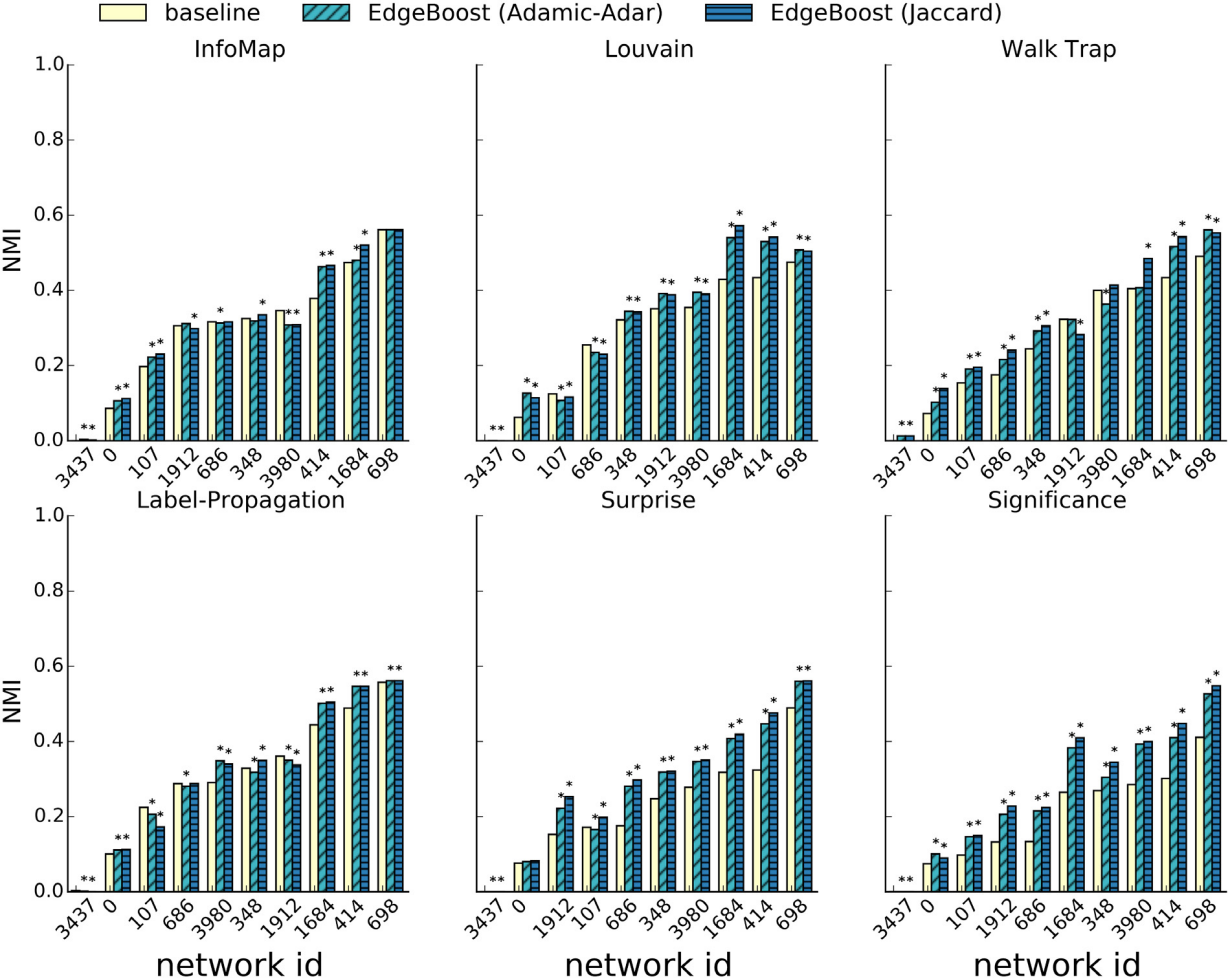
Performance of EdgeBoost on Facebook Networks. Comparison of EdgeBoost on ego-networks from Facebook.

The ground-truth for the Facebook ego-networks can contain overlapping communities, therefore we cannot directly use the standard version of NMI for evaluation. To test EdgeBoost on overlapping ground-truth data we use the NMI extension proposed by Lancichinetti *et al*. [44] that supports comparison of overlapping communities. Fig 10 shows the results of using EdgeBoost with the same six community detection algorithms used in the LFR experiments. The solid bars represent the performance of the baseline community detection algorithm without EdgeBoost. The diagonal and horizontal striped bars shows the results from EdgeBoost paired with the Adamic-Adar and Jaccard respectively. We set the number of iterations for EdgeBoost at 50. Each bar was generated by averaging the NMI score over 100 runs of the baseline and EdgeBoost paired with the Jaccard and Adamic-Adar link predictors. EdgeBoost shows an improvement on most networks for each of the six community detection algorithms; this result is consistent with our experiments on the LFR benchmark. On the LFR benchmark networks EdgeBoost paired with Jaccard link prediction was consistently better than the other link prediction methods but this is not consistently the case on the Facebook networks. Jaccard outperforms the Adamic-Adar most of the time, but there are some cases when the opposite is true. While EdgeBoost shows improvement for most combinations of algorithms and network, there are some instances when the performance of EdgeBoost is lower than baseline. Overall, in 52 of the 60 total configurations EdgeBoost improves performance by an average of 21%. In the rare configurations (8 out of 60) when EdgeBoost performs worse than baseline, EdgeBoost performs only 5% worse on average.

### Varying the Parameters of EdgeBoost

**Fig 11:**
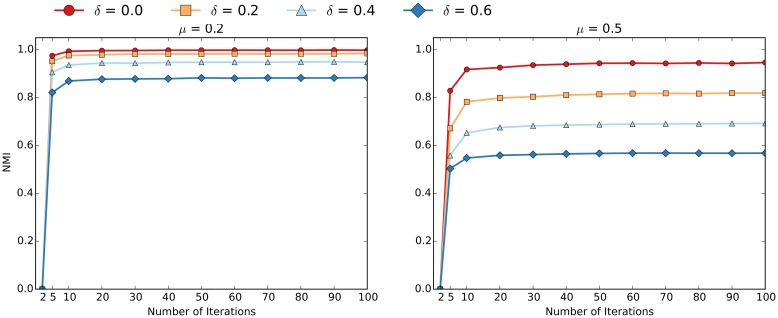
Varying *NumIterations* for EdgeBoost with Louvain. The parameters are set as follows: *μ* = 0.2 (left) and *μ* = 0.5 (right) over *δ* values ranging from 0.0 to 0.6.

**Fig 12:**
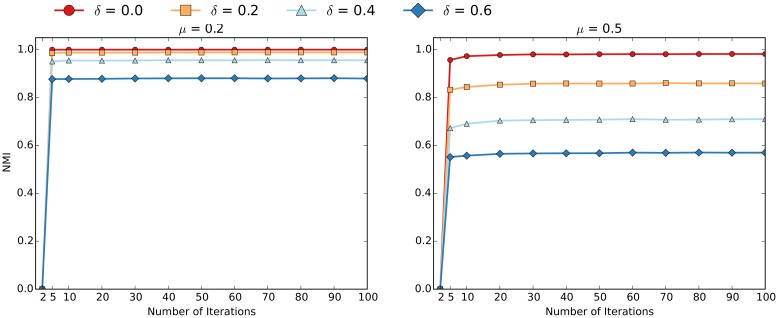
Varying *NumIterations* for EdgeBoost with InfoMap. The parameters are set as follows: *μ* = 0.2 (left) and *μ* = 0.5 (right) over *δ* values ranging from 0.0 to 0.6.

In addition to comparing EdgeBoost using different community detection algorithms we also analyzed how the performance varies with respect to different parameter settings. For these experiments, the curves were generated by averaging over 50 networks generated via the LFR benchmark. Figs 11 and 12 show the convergence of the Louvain and InfoMap algorithms, as a function of the “number of community detection iteratations” (*NumIterations*). Most of the performance gain from EdgeBoost can be had with *NumIterations* set to 10, and setting the number of iterations beyond 50 does not give much benefit. The convergence of EdgeBoost is qualitatively similar for low and high values of *μ* and the entire range of *δ* values.

**Fig 13:**
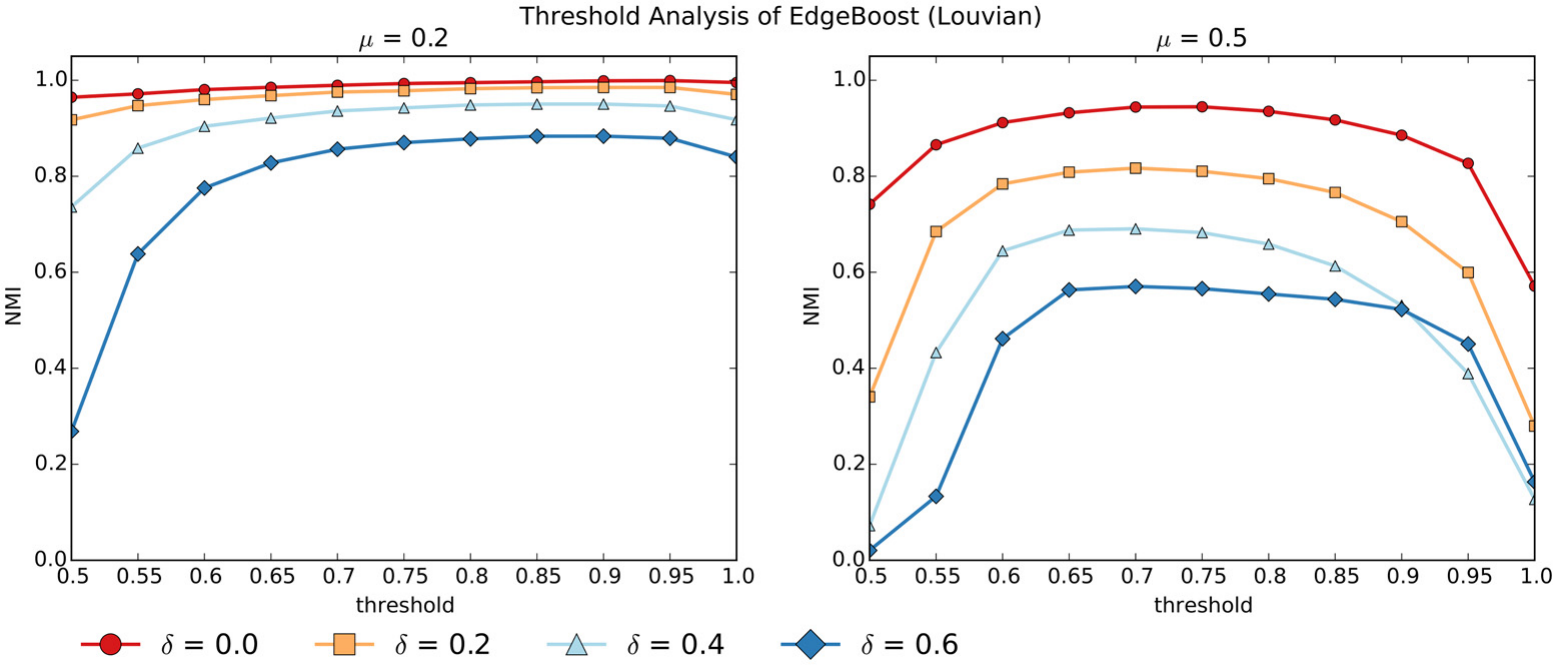
Varying *τ* for EdgeBoost with Louvain. Varying the co-community threshold (*τ*) for EdgeBoost with *μ* = 0.2 (left) and *μ* = 0.5 (right) over *δ* values ranging from 0.0 to 0.6.

**Fig 14:**
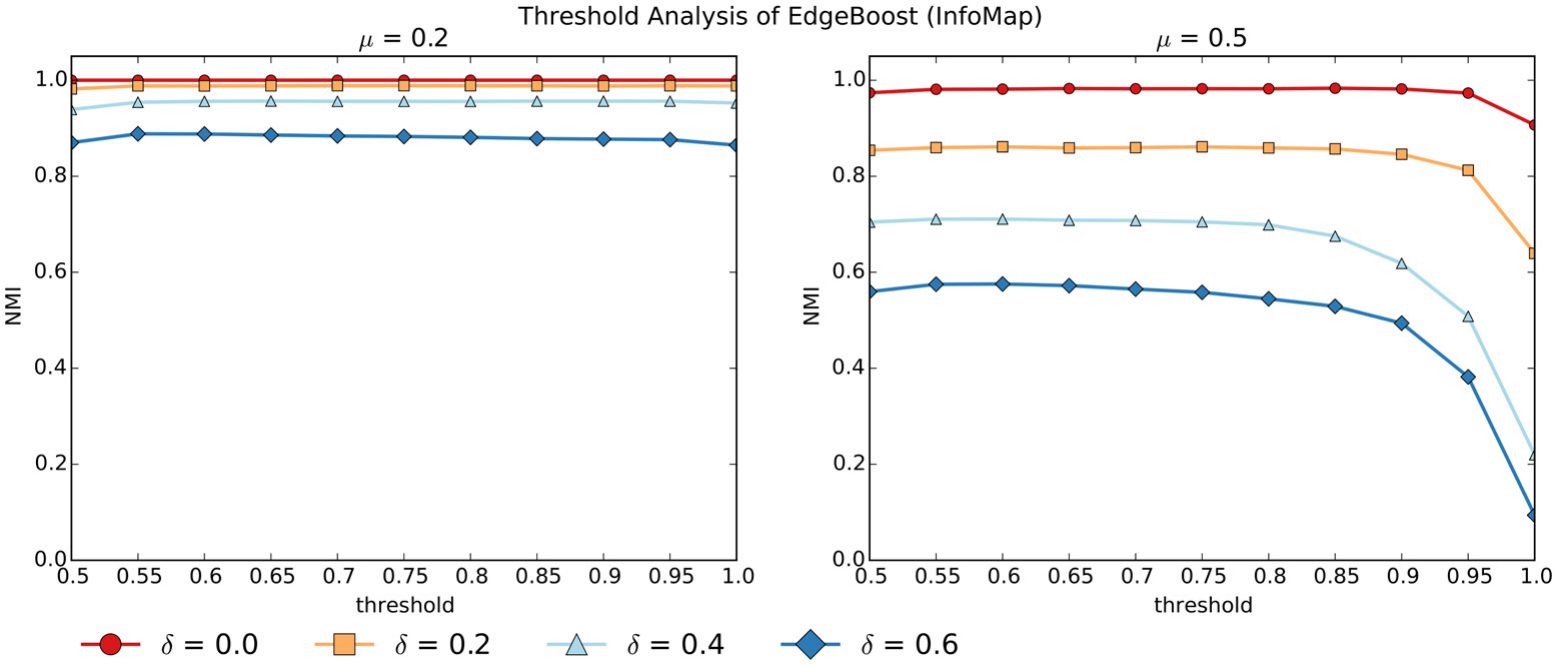
Varying *τ* for EdgeBoost with InfoMap. Varying the co-community threshold (*τ*) for EdgeBoost with *μ* = 0.2 (left) and *μ* = 0.5 (right) over *δ* values ranging from 0.0 to 0.6.

In the *Partition Aggregation* section we propose a method for automatically selecting the co-community threshold *τ*, which we have used for all of the previous experiments. Since the selection of *τ* is the most computationally expensive part of the entire EdgeBoost pipeline, we present an analysis of how EdgeBoost performs with a manual selection of *τ*. Figs 13 and 14 show how EdgeBoost performs by varying the selection of *τ* for EdgeBoost paired with Louvain and InfoMap respectively. For both algorithms, EdgeBoost can achieve good performance for values of *τ* in the range 0.6-0.9, indicating that manual *τ* selection can be an effective way to save computational resources and still boost performance over baseline. For higher values of *μ*, the performance of EdgeBoost is more dependent on *τ*, especially for the Louvain algorithm. Since the Louvain algorithms performs less reliably for higher *μ* values, the co-community network has noisier edge weights, therefore making the selection of *τ* more critical to achieving good performance.

In conclusion, if the user is computationally constrained, simply selecting a manual threshold (or a few thresholds) can give good results without requiring the costly step of computing connected components at each threshold. A potential pitfall of manually selecting a threshold, is that EdgeBoost can give degenerate solutions. Degenerate partitions—those that put all nodes in one cluster or creating hundreds of small clusters—result from the threshold value being too small or large respectively. For applications with a user in the loop, these degenerate solutions are easily detected and fixed by increasing or decreasing the threshold. The automatic threshold finder is intended for applications where full automation is required.

### Runtime Analysis

**Fig 15:**
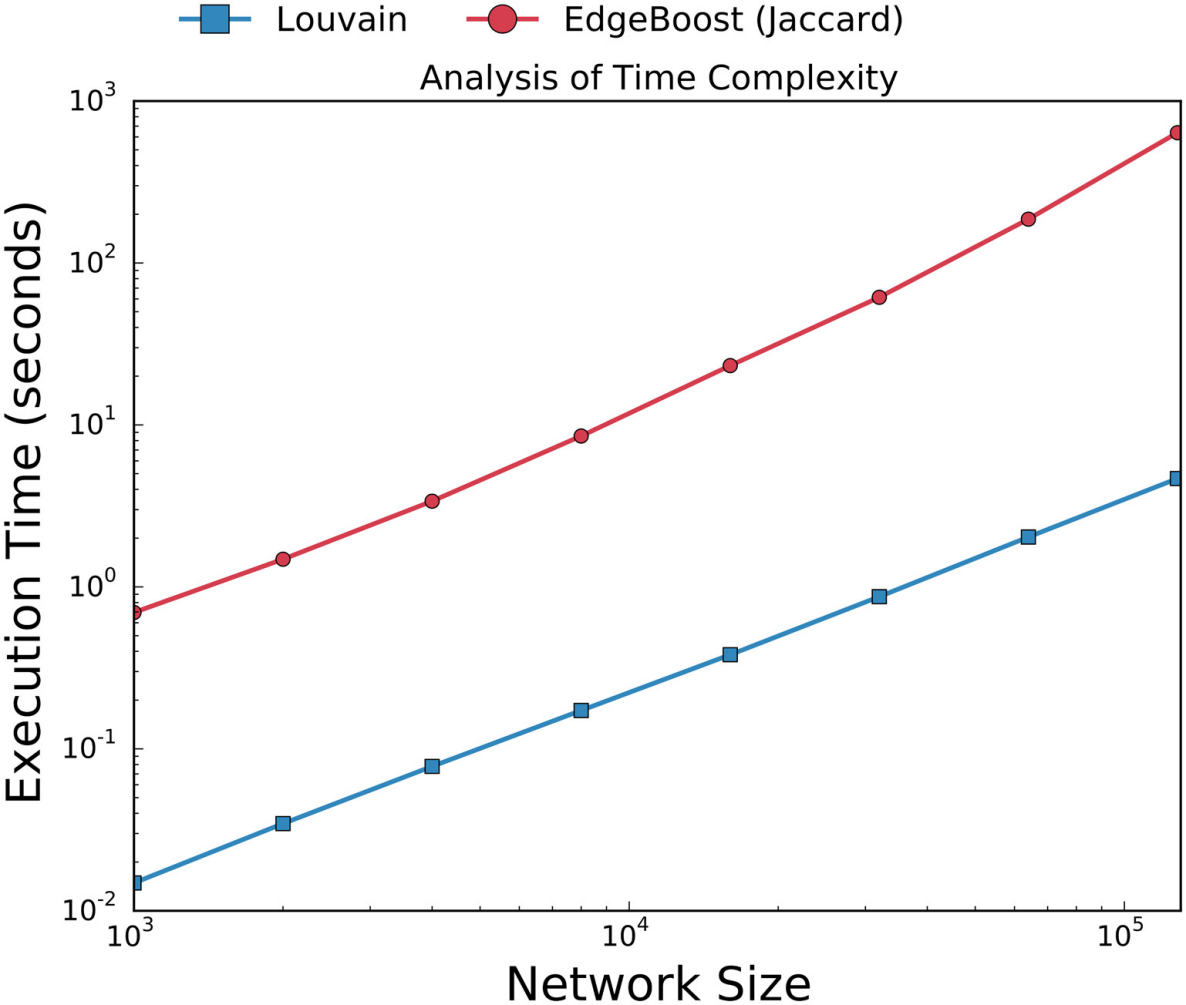
Analysis of Execution Time. Comparison of the runtime between EdgeBoost and baseline Louvain algorithm on networks ranging from size 1000 to 128000 nodes. EdgeBoost has the *NumIterations* set to 50.

The most computationally expensive module of EdgeBoost is the aggregation algorithm which requires the computation of connected components at various thresholds. The complexity of computing connected components is worst case *O*(|*E*|), where |*E*| is the number of edges in the network. The aggregation module computes the connected components on the co-community network, which can be much denser than the input network. In theory it is possible for the co-community network to have *O*(*n*^2^) number of edges, therefore making the aggregation module computationally expensive. In order to show that EdgeBoost scales well when increasing to large networks we ran it on LFR networks of various sizes, ranging from 1000 to 128000 nodes. Fig 15 shows the run time of EdgeBoost with Louvain and Jaccard link prediction with the number of iterations set to 10. While EdgeBoost does have a significant time overhead over Louvain, it still scales in the same manner as Louvain. There are also many components of EdgeBoost’s pipeline that can be naively parallelized. The creation and clustering of the imputed networks are all independent of each other and can be done in parallel. In addition the process of identifying the *τ* threshold can also be sped up by finding the connected components for various threshold values in parallel.

## Discussion and Future Work

As shown in our experiments, EdgeBoost is able to make bigger improvements for certain community detection methods than for others. Our hypothesis for why EdgeBoost works better for certain algorithms has to do with the type of objective functions used by a given method. Objective functions such as modularity are less robust to missing edges in a network because the presence of direct links between nodes in a community are computed directly in the objective. In contrast, the MAP objective function used by the InfoMap algorithm relies on evaluating the community structure based on random walks, and is therefore less affected by direct links between nodes in a community. Our hypothesis is that objectives such as the MAP equation are harder to improve with EdgeBoost because their objectives are already more robust to missing edges. Despite the differences between objective functions, EdgeBoost is still able to show an improvement for most community detection methods for both LFR benchmark and real-world networks.

Our modified LFR benchmark used random edge deletion to model missing edges in networks. While we chose this model because we think that it is the most generally applicable, there are other possibilities for modeling missing edges. One area of future research is to see how community detection algorithms are affected using different edge deletion strategies. Further experiments are necessary to determine if our techniques withstand biased edge removal, but we believe that repeated link prediction will nonetheless boost performance. Further, if missing intra-community edges could be modeled more accurately, development of better link prediction algorithms for enhancing community detection may be possible.

In order to increase the quality of community detection, EdgeBoost trades off time and space efficiency. The construction of the co-community network can be memory intensive because it is likely to be much denser than the input network. In addition, EdgeBoost requires many runs of a sometimes costly community detection algorithm. While EdgeBoost can scale to reasonably large networks (see the *Runtime Analysis* section), we acknowledge these trade-offs and emphasize that EdgeBoost is not designed for million node networks. Instead it was designed for use on small and medium networks (i.e., ego-networks, citation networks), in which data sparsity problems are common, and communities reflect meaningful structures in the data.

While we have shown the efficacy of EdgeBoost in computing better partitions, it is possible that the approach can also improve other types of community analysis. Given different thresholds for which we can prune the co-community network (see the *Partition Aggregation* section) and the corresponding set of connected components, we can obtain a set of communities with a specified confidence. Some applications may not require a complete partitioning of nodes and may even be better suited with an incomplete partition which has higher quality communities. In future work we would also like to see how EdgeBoost can be used in the detection of overlapping and/or hierarchical communities. This extension would require a different aggregation function as our current method is only capable of creating strict partitions, via computing connected-components.

The link-predictors tested in this paper are all based on shared neighbors, and therefore are only capable of inferring missing connections between nodes that are at maximum 2 hops from each other. One issue with predicting links that are further apart is the computational complexity, since most of the metrics that are not neighborhood based are based off the number of shortest paths between pairs of nodes. While not presented in this paper, we experimented with the local path index proposed by [45], which predicts links between nodes that are as far as 3 hops from each other, but did not see any noticeable improvement. Other link predictors that we did not explore are those that utilize node attributes (*e.g*., school and city) and/or link structure to score missing edges. Since most of the methods in disjoint community detection do not account for node attributes, in the future EdgeBoost could be a robust way to integrate node attributes into existing algorithms.

## Conclusions

Networks inferred or collected from real data are often susceptible to missing edges. We have shown that as the percentage of missing edges in a network grows, the quality of community detection decreases substantially. To counter this, we proposed EdgeBoost as a framework to improve community detection on incomplete networks. EdgeBoost is capable of improving all the community detection algorithms we tested with its novel application of repetitive link prediction, on real ego-networks from Facebook. EdgeBoost is an easy-to-implement meta-algorithm that can be used to improve any user-specified community detection algorithm and we anticipate that it will be useful in many applications.

## Supporting Information

S1 Fig NMI heat map of six community detection algorithms. the parameters *μ* and *δ* are represented on the *x* and *y* axis respectively. Each square is labeled with the corresponding NMI value.
(TIF)

S2 Fig RE heat map of six community detection algorithms. the parameters *μ* and *δ* are represented on the *x* and *y* axis respectively. Each square is labeled with the corresponding RE value.
(TIF)

S3 Fig Distribution of community sizes for Facebook ego networks. Nodes were given community labels by ego users as part of a user study.
(TIF)

S4 Fig EdgeBoost Paired With InfoMap. Performance of EdgeBoost (solid) and the baseline InfoMap algorithm (dashed) on LFR benchmarks. The purple shaded region shows the improvement of EdgeBoost for NMI. The of plots shows the relative error of the partition size.
(TIF)

S5 Fig EdgeBoost Paired With WalkTrap. Performance of EdgeBoost (solid) and the baseline WalkTrap algorithm (dashed) on LFR benchmarks. The purple shaded region shows the improvement of EdgeBoost for NMI. The bottom row shows the relative error of the partition size.
(TIF)

S6 Fig EdgeBoost Paired With Surprise. Performance of EdgeBoost (solid) and the baseline Surprise algorithm (dashed) on LFR benchmarks. The purple shaded region shows the improvement of EdgeBoost for NMI. The bottom row shows the relative error of the partition size.
(TIF)

S7 Fig EdgeBoost Paired With Significance. Performance of EdgeBoost (solid) and the baseline Significance algorithm (dashed) on LFR benchmarks. The purple shaded region shows the improvement of EdgeBoost for NMI. The bottom row shows the relative error of the partition size.
(TIF)

S8 Fig EdgeBoost Paired With Label-Propagation. Performance of EdgeBoost (solid) and the baseline Label-Propagation algorithm (dashed) on LFR benchmarks. The purple shaded region shows the improvement of EdgeBoost for NMI. The bottom row shows the relative error of the partition size.
(TIF)
