# Supplementary figures and images for: Link-Prediction Enhanced Consensus Clustering for Complex Networks
Source: PLoS One. 2016 May 20;11(5):e0153384. doi: 10.1371/journal.pone.0153384 (PMC4874693; doi:10.1371/journal.pone.0153384)

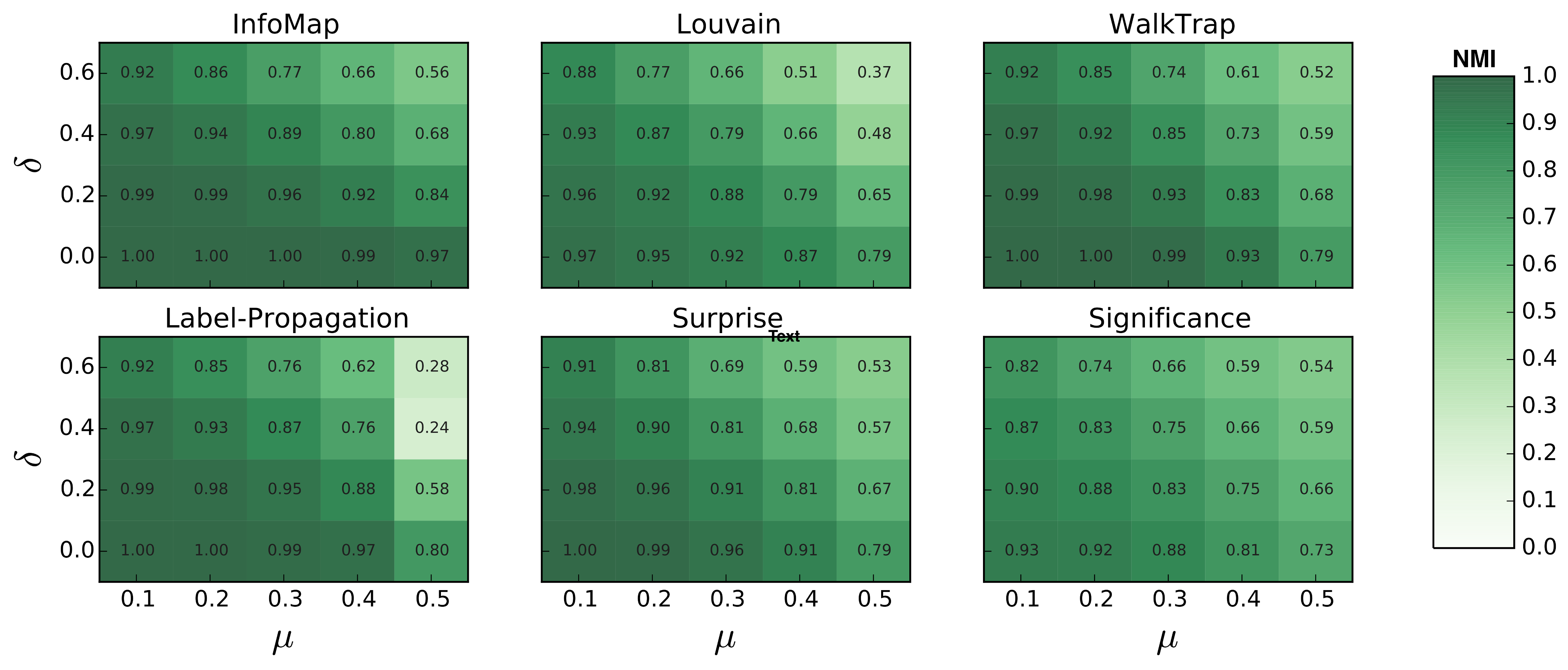

Supplement: S1 Fig — the parameters μ and δ are represented on the x and y axis respectively. Each square is labeled with the corresponding NMI value. (TIF) [file pone.0153384.s001.tif]

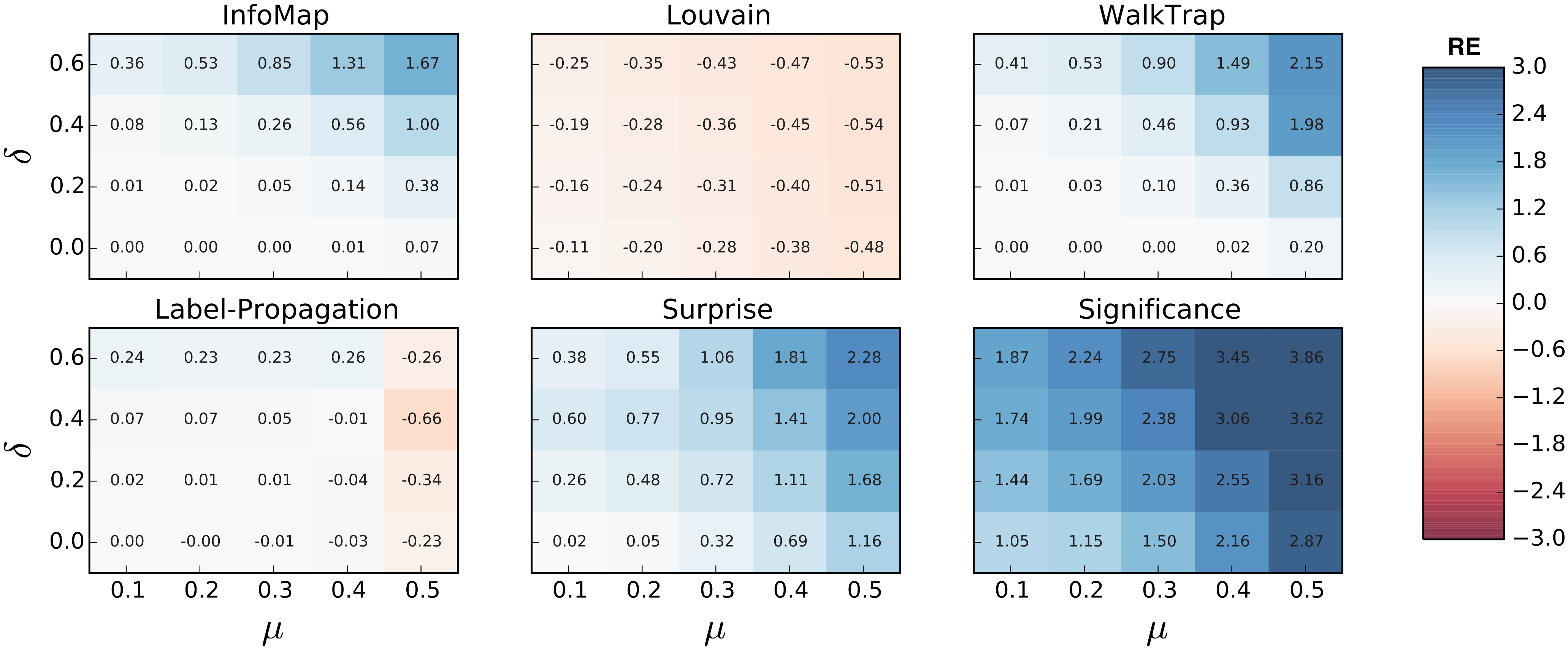

Supplement: S2 Fig — the parameters μ and δ are represented on the x and y axis respectively. Each square is labeled with the corresponding RE value. (TIF) [file pone.0153384.s002.tif]

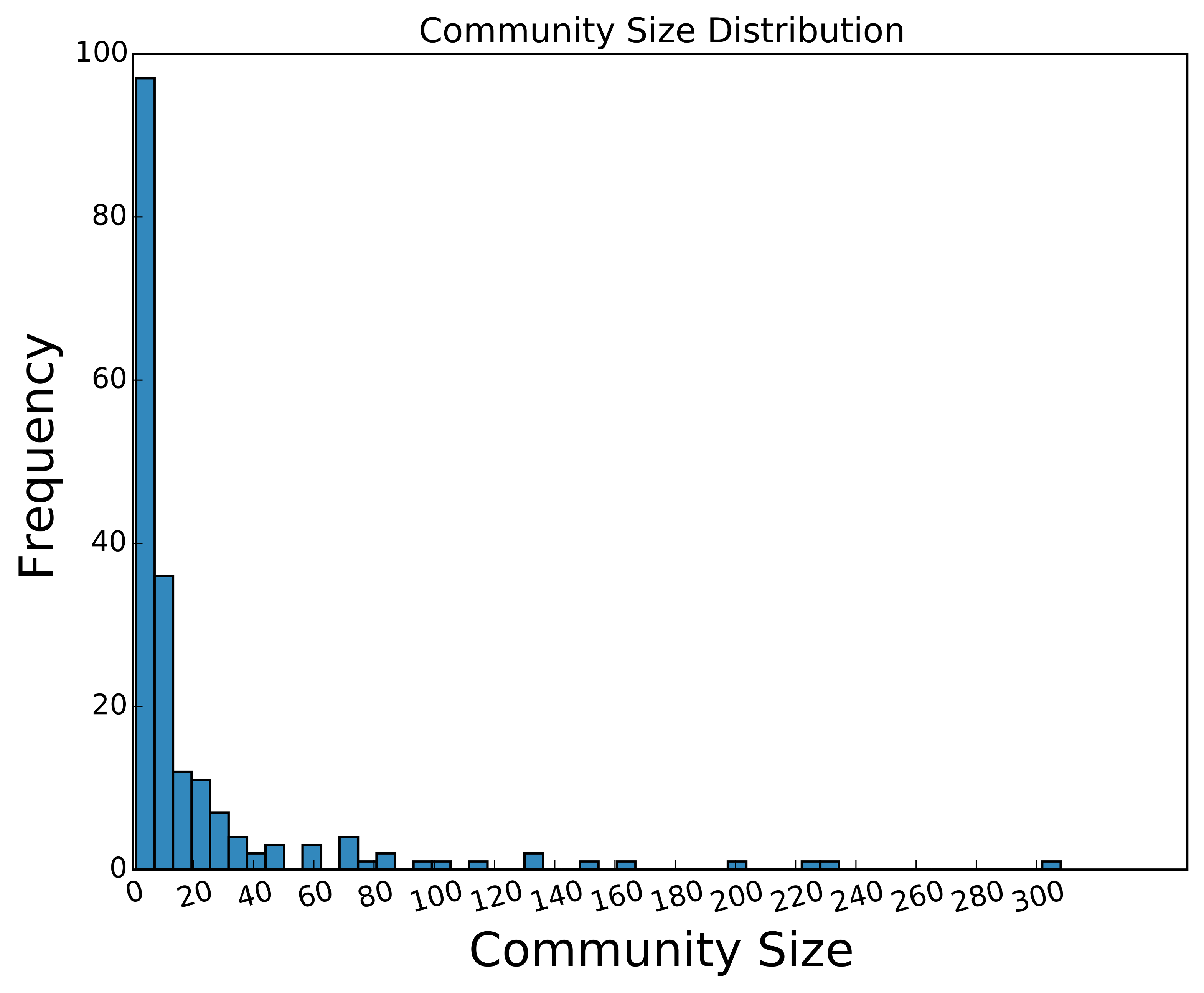

Supplement: S3 Fig — Nodes were given community labels by ego users as part of a user study. (TIF) [file pone.0153384.s003.tif]

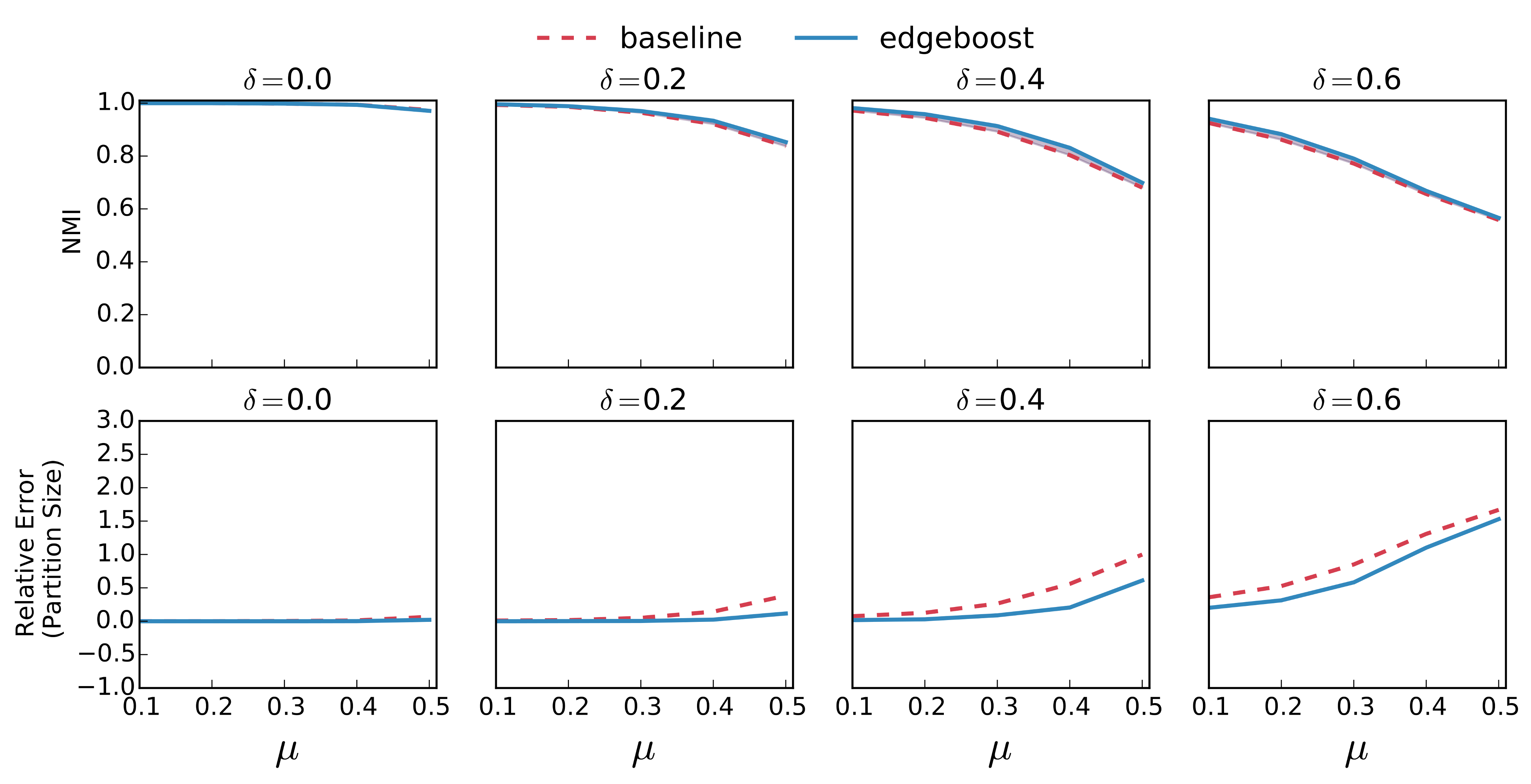

Supplement: S4 Fig — Performance of EdgeBoost (solid) and the baseline InfoMap algorithm (dashed) on LFR benchmarks. The purple shaded region shows the improvement of EdgeBoost for NMI. The of plots shows the relative error of the partition size. (TIF) [file pone.0153384.s004.tif]

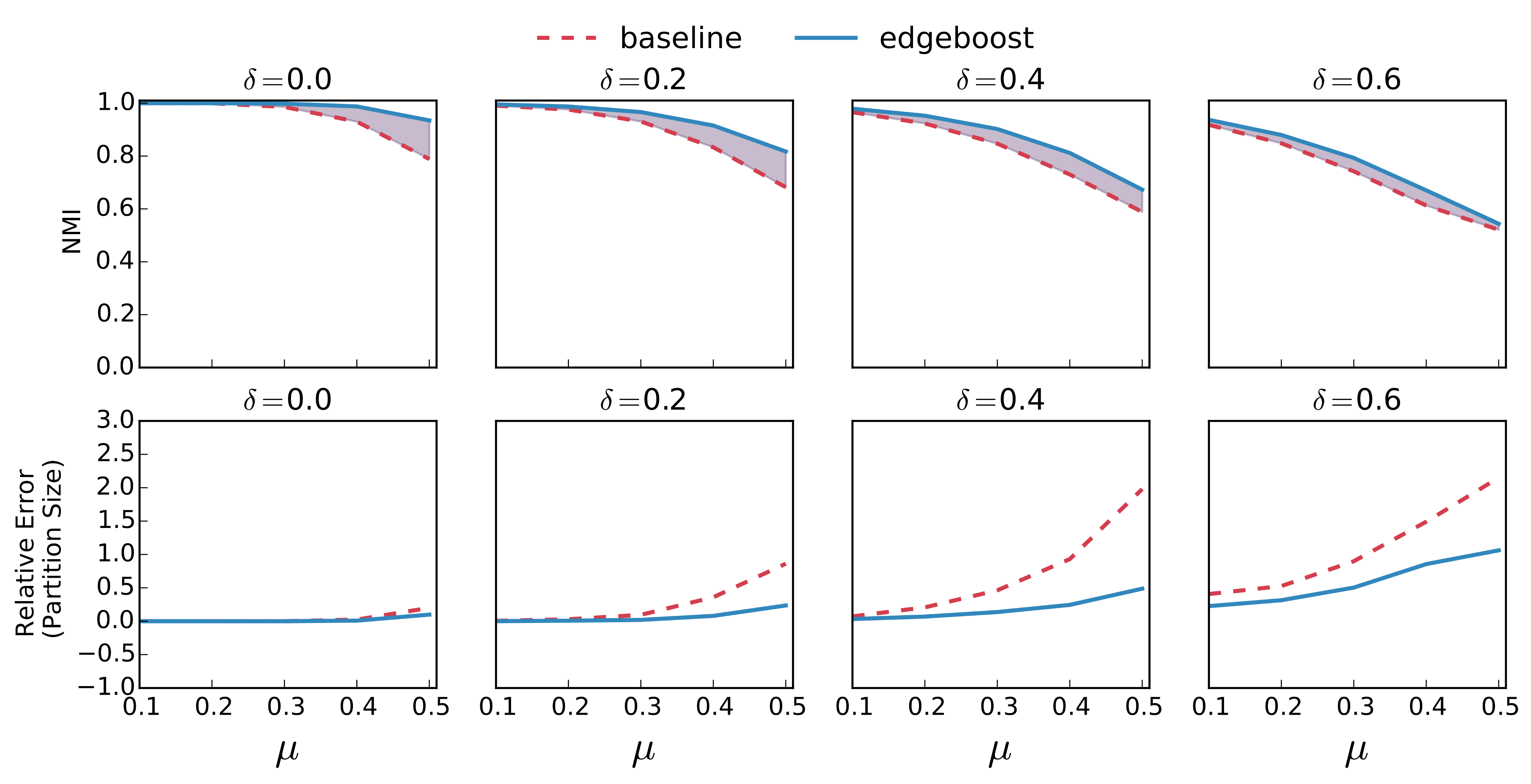

Supplement: S5 Fig — Performance of EdgeBoost (solid) and the baseline WalkTrap algorithm (dashed) on LFR benchmarks. The purple shaded region shows the improvement of EdgeBoost for NMI. The bottom row shows the relative error of the partition size. (TIF) [file pone.0153384.s005.tif]

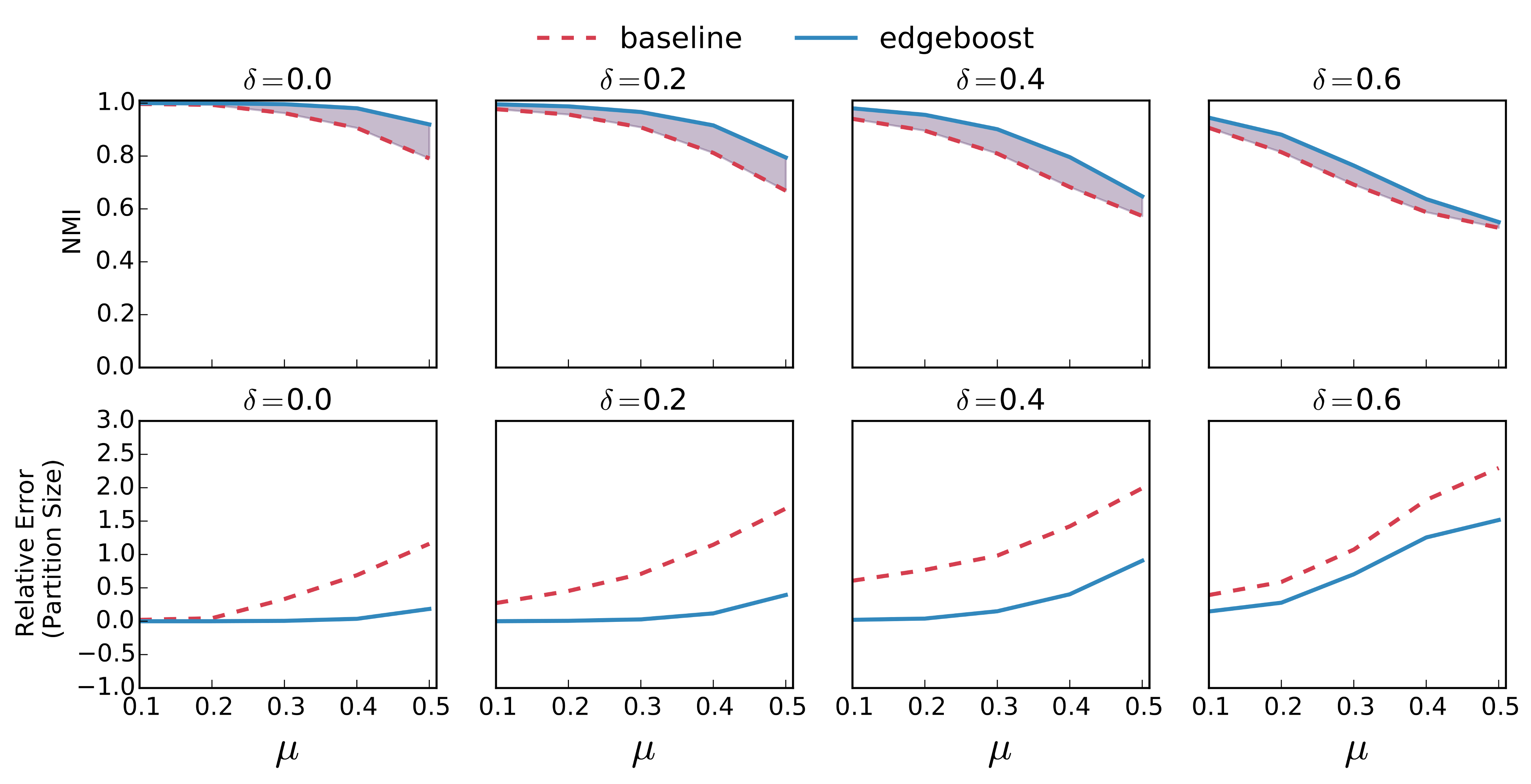

Supplement: S6 Fig — Performance of EdgeBoost (solid) and the baseline Surprise algorithm (dashed) on LFR benchmarks. The purple shaded region shows the improvement of EdgeBoost for NMI. The bottom row shows the relative error of the partition size. (TIF) [file pone.0153384.s006.tif]

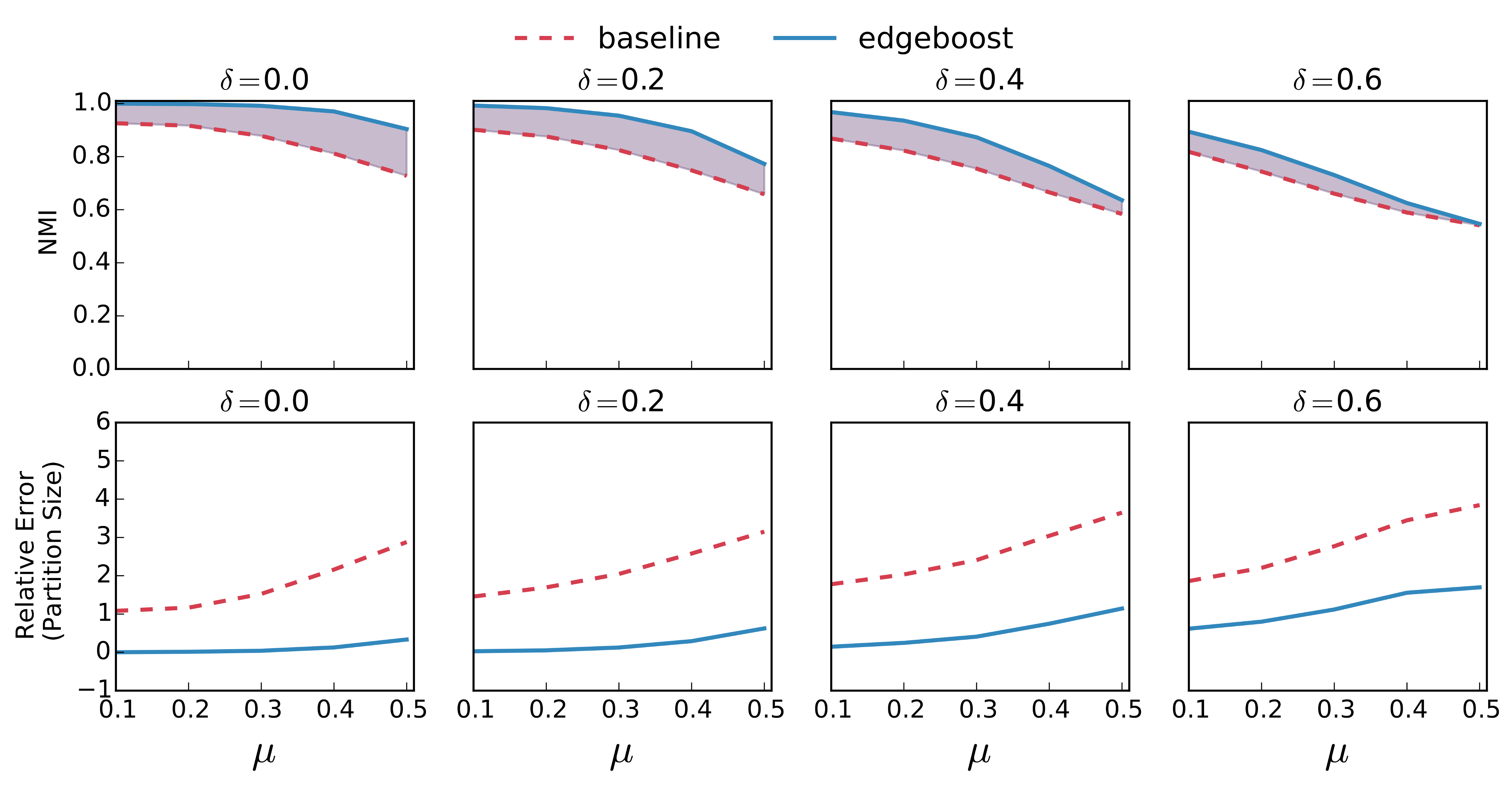

Supplement: S7 Fig — Performance of EdgeBoost (solid) and the baseline Significance algorithm (dashed) on LFR benchmarks. The purple shaded region shows the improvement of EdgeBoost for NMI. The bottom row shows the relative error of the partition size. (TIF) [file pone.0153384.s007.tif]

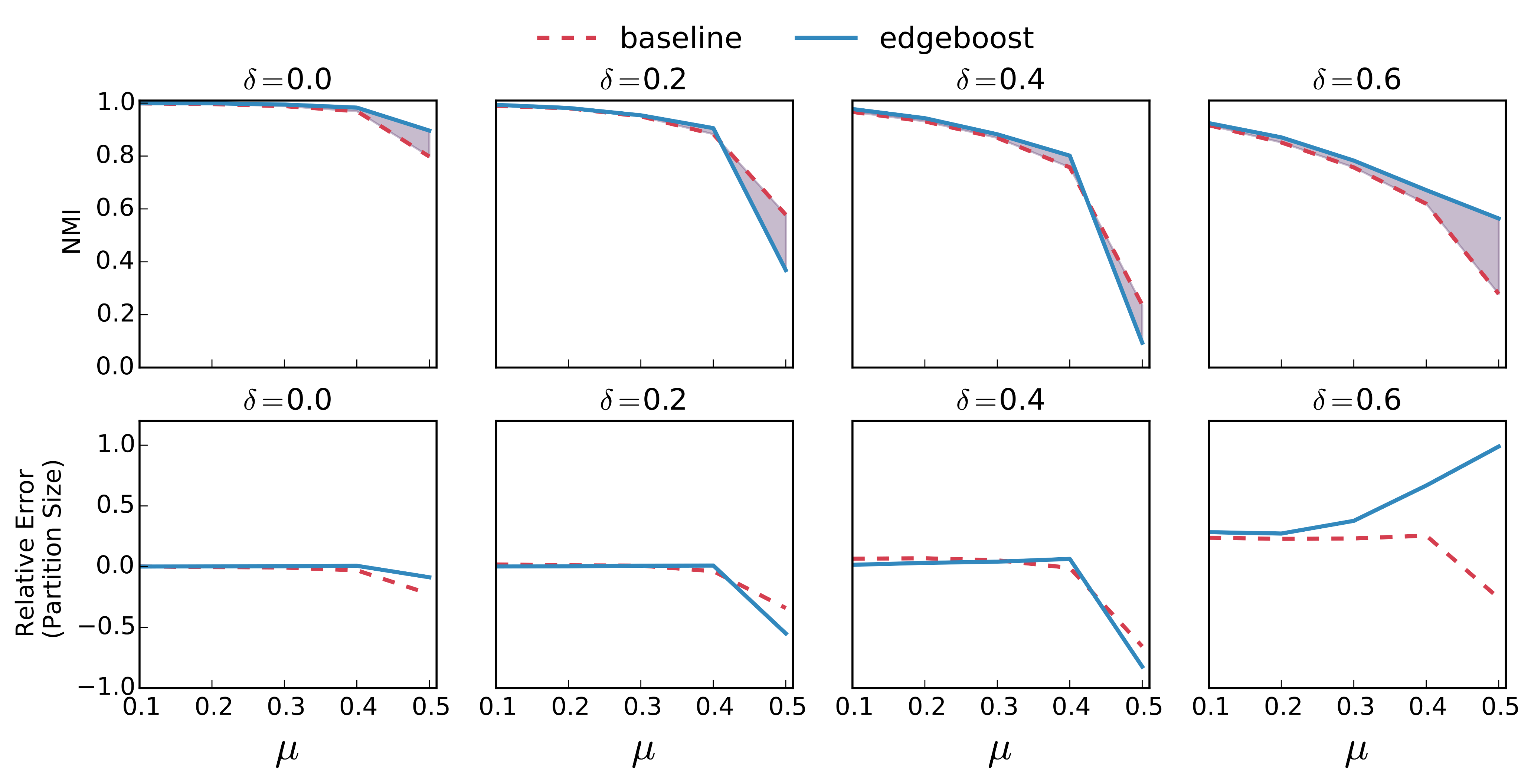

Supplement: S8 Fig — Performance of EdgeBoost (solid) and the baseline Label-Propagation algorithm (dashed) on LFR benchmarks. The purple shaded region shows the improvement of EdgeBoost for NMI. The bottom row shows the relative error of the partition size. (TIF) [file pone.0153384.s008.tif]
